# *Dioscorea* Zingiberensis New Saponin Inhibits the Growth of Hepatocellular Carcinoma by Suppressing the Expression of Long Non-coding RNA TCONS-00026762

**DOI:** 10.3389/fphar.2021.678620

**Published:** 2021-05-03

**Authors:** Xing Liu, Pingsheng Zhou, Keqing He, Zhili Wen, Yong Gao

**Affiliations:** ^1^ Department of Oncology, Shanghai East Hospital, Tongji University School of Medicine, Shanghai, China; ^2^ Department of Medicine, Jinggangshan University, Ji’an, China; ^3^ International Education College, Jiangxi University of Traditional Chinese Medicine, Nanchang, China; ^4^ Department of Hepatobiliary Diseases, Affiliated Hospital of Jiangxi University of Traditional Chinese Medicine, Nanchang, China; ^5^ Department of Gastroenterology, The Second Affiliated Hospital of Nanchang University, Nanchang, China

**Keywords:** Hepatocellular carcinoma (HCC), long non-coding RNA (lncRNA), dioscorea zingiberensis new saponin (ZnS), TCONS-00026762, apoptosis

## Abstract

**Background:** The etiology and carcinogenesis of hepatocellular carcinoma (HCC) are associated with various risk factors. Saponins extracted from *Dioscorea* zingiberensis C. H. Wright exhibit antitumor activity against HCC. This study aimed to investigate the effect and the underlying mechanism of *Dioscorea* Zingiberensis new saponin (ZnS) on HCC.

**Methods:** Human HCC cell lines, Huh7 and SMMC-7721, were treated with different concentrations of ZnS. Cell apoptosis was determined *via* flow cytometry assay. Differentially expressed lncRNAs (DElncRNAs) in ZnS-treated SMMC-7721 cells were determined through RNA-sequence. The role of lncRNA TCONS-00026762 in HCC was investigated gain of function analysis, along with cell proliferation, apoptosis, and invasion in HCC cells. A subcutaneous xenograft of SMMC-7721 cell lines was established to study the effects of TCONS-00026762 *in vivo*. The expression of apoptosis-related proteins was detected *in vivo* and *in vitro via* western blotting.

**Results:** ZnS inhibited the proliferation of HCC cell in a dose-dependent manner. ZnS could induce apoptosis in HCC cells. Illumina sequencing results showed that 493 DElncRNAs were identified in ZnS-treated SMMC-7721 cells. TCONS-00026762 expression was down-regulated in the ZnS-treated SMMC-7721 cells. TCONS-00026762 inhibited the effect of ZnS on the proliferation, apoptosis, and invasion of HCC cells. ZnS inhibited the tumor growth, while, TCONS-00026762 promoted tumor growth *in vivo*. Furthermore, ZnS and TCONS-00026762 regulated cell apoptotic pathways.

**Conclusion:** ZnS significantly inhibits the viability, apoptosis, invasion, and tumorigenicity of HCC cells by regulating the expression of TCONS-00026,762. Our findings provide novel insights into the potential role of lncRNA in HCC therapy.

## Introduction

Hepatocellular carcinoma (HCC) is among top 10 cancers that exhibit high mortality rates worldwide. Over 840,000 new cases of HCC are reported and 780,000 people die of HCC annually (Ferlay et al., 2015; Bray et al., 2018; Yu et al., 2021). Around the world, the leading cause of liver cancer is chronic viral hepatitis, namely hepatitis B and C. However, due to the increase in hepatitis B vaccination, studies have predicted that the rising incidence of alcohol consumption and obesity worldwide will overtake viral hepatitis as the most common cause of HCC (Akinyemiju et al., 2017). And alcohol (ethanol) is already the most common cause of liver cancer in men (Akinyemiju et al., 2017), recognized as a Group 1 carcinogen by the International Agency for Research on *Cancer* (IARC) (Byrne and Targher, 2015). Alcohol is mainly detoxified and metabolized by the liver, and the intermediate product of metabolism, acetaldehyde, has a direct toxic effect on liver cells. Long-term intake of large amounts will undoubtedly increase the burden on the liver and accelerate liver damage, and alcohol can stimulate the secretion of the pituitary gland, accelerate the speed of cell division, increase the susceptibility to cancer, and thus easily induce alcoholic liver, alcoholic cirrhosis, and even liver cancer (Dunn and Shah, 2016). Due to the difficulty of early diagnosis, rapid disease development, and lack of targeted medicines, the survival rate of hepatocellular carcinoma is very limited (Fu and Wang, 2018). Most patients presenting with very advanced HCCs are untreatable and die within 3–6 months (Ferlay et al., 2015). Hence, it is important to explore new biomarkers and evaluate effective agents for diagnosis and treatment of HCC.

Long non-coding RNA (lncRNA) is a non-coding RNAs exceeding 200 nucleotides (nt) in length. Recent studies have confirmed that lncRNA plays a potentially complex and diverse role in the etiology, carcinogenesis, invasion, and metastasis of human cancers, including HCC (Yuan et al., 2014; Wang et al., 2015; Li et al., 2016). LncRNA regulates the expression of tumor suppressor/anticancer genes, mainly by sponging miRNAs and regulating mRNAs (Wang et al., 2015; Li et al., 2016; Lei et al., 2019). For example, Li et al., reported that long non-coding RNA HULC (highly up-regulating in liver cancer) expression activated ZEB1 (Zinc finger E-box binding homeobox 1)-induced epithelial-mesenchymal transition (EMT) by sponging miR-200a-3p (Bray et al., 2018). Long noncoding RNA NR2F1-AS1 (nuclear receptor subfamily 2 group F member 1 antisense RNA 1) and ARSR (Activated in RCC with sunitinib Resistance) are associated with drug resistance in HCC cells *via* modulation of signaling pathways and miRNA or mRNAs (Li et al., 2017; Huang et al., 2018). Accordingly, the dysregulation of lncRNA in HCC is associated with diverse biological processes in HCC cells, such as cell differentiation, proliferation, invasion, metastasis, and apoptosis (Li et al., 2015; Yang et al., 2017).

Some signal transductions mediated by lncRNA are closely related to the anticancer mechanism of various drugs. It has been reported that saponins extracted from plants belongs to the family Dioscoreaceae, including *Dioscorea* zingiberensis C. H. Wright, exhibit antitumor activity against HCC (Zhou et al., 2009; Zhao et al., 2018). D. zingiberensis C. H. Wright is rich in saponins, including protodioscin, dioscin, scutellaria, trifoliate, glucose triglucoside, glucose diglucoside and trillium glucoside (Zhu et al., 2010; Lei et al., 2011; Zhang et al., 2015). Saponins induce apoptosis and cell cycle arrest *via* the extracellular regulated protein kinase (ERK)/nuclear factor κB (NF-κB) signaling pathway in human HCC HepG2 cells (Auyeung et al., 2009; Long et al., 2015). Following, Qiu et al. confirmed that Rhizoma Paridis saponins through inhibiting cancer cellular metabolism to suppress proliferation in hepatoma H22 tumor murine (Qiu et al., 2016). In addition, Rhizoma Paridis saponin treatment reversed sorafenib-resistance in HCC cells (Yao et al., 2018). D. zingiberensis C. H. Wright, also known as turmeric, is a perennial herbaceous plant of the genus *Dioscorea* and a unique species cultivated in China. The main active ingredient in the rhizome of D. zingiberensis is a saponin, which displays antitussive, antiasthmatic, antithrombostic, and cholesterol lowering activities, in addition to preventing arteriosclerosis (Gong et al., 2011; Li et al., 2011; Du et al., 2016). The results of our previous study indicated that HCC cell proliferation was significantly reduced when treated with Zingiberensis new saponin (ZnS) from D. zingiberensis C. H. Wright. However, the mechanism underlying of antitumor activity of ZnS against HCC cells remains unclear.

**FIGURE 1:**
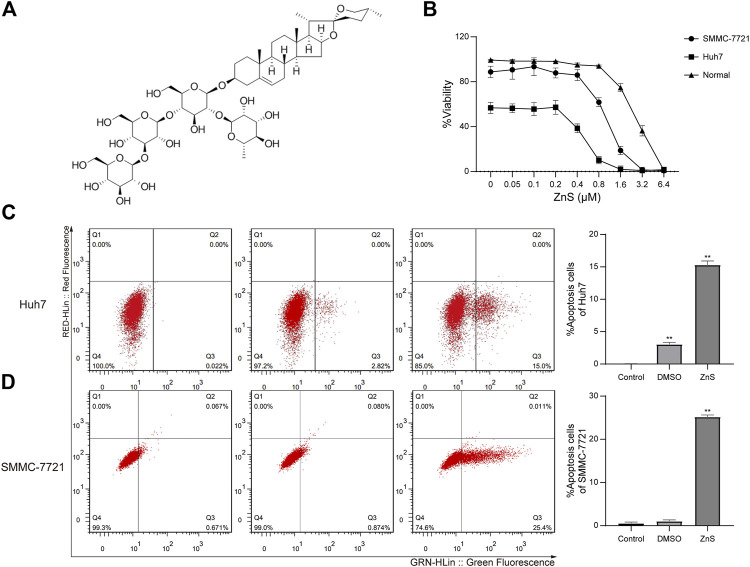
**(A)** The chemical structure of ZnS. **(B)** The viability rates of human normal liver cell line, HL-7702, and human HCC cell lines, Huh7 and SMMC-7721, treated with ZnS, were examined using CCK-8. Detection of apoptosis rates in two cell lines, Huh7 **(C)** and SMMC-7721 **(D)**, after ZnS treatment by flow cytometry. Each trial was replicated at least three times. Data are expressed as mean ± SD, **, *p* < 0.01.

In this study, we demonstrated that ZnS suppresses proliferation of HCC cells by inhibiting the expression of lncRNA TCONS-00026,762. The chemical structure of ZnS is shown in Figure 1A. We determined the differentially expressed lncRNAs by identifying the lncRNA profiles in HCC cells treated with ZnS. Overexpression of TCONS-00026762, *via* transfection into HCC cells, was assessed in order to investigate mechanisms underlying in HCC. ZnS and TCONS-00026762 regulated the proliferation of HCC cells and tumor growth by inducing cell apoptosis. Moreover, the expression of apoptosis-related proteins was examined, and it was demonstrated that ZnS, a naturally occurring phytochemical agent, inhibited HCC by regulating TCONS-00026762.

## Material and Methods

### Cell Lines, Culture Conditions, Plasmids, and Reagents

Human normal liver cell line, HL-7702, and human HCC cell lines, Huh7 and SMMC-7721, were purchased from the Chinese Academy of Sciences (Beijing, China). All cells were maintained in Dulbecco’s modified Eagle’s medium (DMEM, Hyclone, Thermo Scientific, Epsom, United Kingdom) supplemented with 10% fetal bovine serum (FBS, Hyclone) and 1% penicillin-streptomycin (Hyclone) at 37°C in 5% CO_2_. Full-length lncRNA TCONS-00026762 was amplified *via* PCR and then subcloned into the vector, pCDH-CMV-MCS-EF1-copGFP-T2A-Puro.

### Cell Treatment

Cells were grown in 96-well plates (5 × 103 cells/well) and treated with ZnS (S12537, HPLC ≥98%, C51H82O22, Shanghai Hewu Biotechnology Co., Ltd., Shanghai, China) at concentrations of 0.05, 0.1, 0.2, 0.4, 0.8, 1.6 3.2, and 6.4 μM for 48 h. Cells treated with DMSO were regarded as the control. IC50 values of ZnS in the two HCC cell lines were detected using a cell viability assay.

### Cell Viability Assay

After being treated with 48 h ZnS, cells were harvested, and the cell viability was assessed using a Cell Counting Kit 8 (CCK-8, Dojindo Laboratories, Kumamoto, Japan), according to the manufacturer’s instructions. Optical density at 450 nm absorbance was detected using a microplate spectrophotometer (Multiskan GO, Thermo Scientific). Six replicates were performed for each experiment. The IC50 values of ZnS in the two cell lines were also calculated.

### Cell Apoptosis Assay

Cell apoptosis was analyzed by Annexin V-fluorescein isothiocyanate (FITC) and PI (Beyotime Institute of Biotechnology). Cells treated with ZnS for 48 h were harvested *via* centrifugation at 1,200 rpm/min for 1 min, and suspended in 1× binding buffer. The cells were then incubated with Annexin V-FITC solution in the dark at 4°C for 10–15 min, followed by a second harvesting (centrifugation at 1,200 rpm/min and suspension). Cells were then subjected to PI staining for observation *via* a BD FacScanto II flow cytometer (BD Biosciences, San Jose, CA, United States).

### RNA-Sequencing and Computational Analysis

Total RNA was extracted using TRIzol (Invitrogen, Carlsbad, CA, United States). RNA qualification and quantification were conducted using a NanoDrop ND-2100 spectrophotometer (NanoDrop Technologies, Wilmington, DE, United States) and an Agilent 2,100 Bioanalyzer (Agilent Technologies, Palo Alto, CA, United States). Certified samples (RNA Integrity Number >7.0 and OD260/OD280 > 1.8) were subjected to elimination of ribosomal RNA (rRNA), using a Ribo-Minus kit (Thermo Fish, Waltham, MA, United States) and RNA fragmentation, *via* using fragmentation buffer (Ambion, Austin, TX, United States).

First strand cDNA and double strand DNA (dsDNA) were subsequently synthetized using reverse transcriptase (SuperScript III, Invitrogen), random primers, and dNTPs. Thereafter, cDNA samples were purified and ligated to adenylate 3′-ends and sequencing adaptors, followed by purification (XP beads, Beckman Coulter, Brea, CA, United States). Next, dsDNA containing uracil deoxyribonucleotides (U base) was degraded. Finally, PCR enrichment followed by purification was performed to obtain sequencing libraries. An Illumina HiSeq 4,000 pair-end sequencing (2 × 150 bp) platform was used.

### LncRNA Profile Analysis

Reference-based assemblies of three samples in each group were merged using Cuffmerge (version 2.2.1, http://cole-trapnell-lab.github.io/cufflinks/cuffmerge/). Known lncRNAs were identified using the NONCODE database (http://www.bioinfo.org/noncode/). Novel lncRNAs in the assemblies were detected using the following criteria: ≥200 nt in length, Coding Potential Calculator (CPC) score ≤ 0, Coding Potential Assessment Tool (CPAT) probability ≤0.364 and phyloCSF score ≤ −20. Fragment perkilobase of transcript per million fragments mapped (FPKM) values were used to calculate the expression levels of the transcripts. Differentially expressed lncRNAs (DElncRNAs) were identified based on FPKM values under conditions of *p* < 0.05 and |log2FC| >1.

### Quantitative Real-Time PCR (qRT-PCR)

SMMC-7721 cells grown in six-well plates were transfected with plasmids. After 24h, the cells were either treated with 1 μM ZnS for 24 h or left untreated and were then harvested. Total RNA was extracted using TRIzol reagent (Invitrogen, United States) in accordance with the manufacturer’s instructions. Thereafter, cDNA was synthesized from total RNA using a PrimeScript RT reagent kit (Tiangen, China). GAPDH mRNA was used as an internal control. The primer sequences used were as follows. TCONS-00026762 forward primer: 5′-AAT GAG GAG CAG GTT GGA CT-3′, TCONS-00026762 reverse primer: 5′-GAT CAC TTC CTC ACC TGG CT-3'; GAPDH forward primer: 5′-GGT GAA GGT CGG AGT CAA CG-3′, GAPDH reverse primer: 5′-CAA AGT TGT CAT GGA TGA ACC-3'. Finally, the expression was determined using the SYBR@ Green reagent kit (Roche, Indianapolis, IN, United States).

### Construction of Stable Cell Lines

SMMC-7721 cells were infected with lentivirus carrying TCONS-00026,762 or control lentivirus (Igebiotech, Guangzhou, China). The efficacy of TCONS-00026762 overexpression was detected *via* fluorescence analysis.

### Matrigel Invasion Assay

First, 4 × 10^5^ cells were seeded into the upper chamber coated with Matrigel (BD Biosciences, San Diego, CA, United States) and incubated in 200 μL serum-free media. The lower chambers were filled with 600 μL complete medium for 24 h. After removing cells on the upper surface, transwell filters were fixed with paraformaldehyde and stained with crystal violet (RiboBio, Guangzhou, China). The experiment was performed in triplicate. Invading cells were captured using a Motic inverted microscope (AE30, Motic, Hong Kong, China), and the average number of invading cells in three randomly selected fields was calculated.

### Tumor Formation in Nude Mice

This animal study and all animal experiments were approved by the Medical Experimental Animal Care Commission of Shanghai East Hospital (Approval number: 2019-52). Animal experiments were performed in accordance with the National Institutes of Health Guide for the Care and Use of Laboratory Animals. Twenty 4-week-old male BALB/c nude mice were randomly assigned to control group (SMMC-7721 cells), ZnS group (SMMC-7721 cells), ZnS + NC group (SMMC-7721 cells with LV-control) and ZnS + TCONS-00026762 (SMMC-7721 with overexpression-TCONS-00026762). The mice were orally administered ZnS at 60 mg/kg/day. Three weeks later, all mice were sacrificed, and the weight of each tumor was measured. Tumor tissues were integrally stripped out.

### Immunohistochemistry

Paraffin-embedded slides of xenograft tumors from the nude mice were dewaxed with xylene I, II, and III for 10 min each, and 100, 90, 70, and 50% alcohol for 2 min each, followed by washing with PBS three times. Samples were subsequently heated in 0.01 M citrate buffer (pH 6.0), incubated in serum for 30 min at 37°C and finally incubated overnight at 4°C with anti-ki67 antibodies. The slides were then incubated with secondary antibodies for 2 h at 37°C, and washed with PBS. After development with DAB-H2O2 for 10 min at room temperature, slides were observed for staining under a light microscope.

### Western Blotting

Lysis buffer, containing 10% glycerol, 2.3% SDS, 62.5 mM Tris, pH 6.8 150 mM NaCl, 10 mM EDTA, 1 μg/ml eupeptic, 1 μg/ml pepstatin, 5 μg/ml chymostatin, 1 μg/ml aprotinin, and 1 mM phenylmethylsulphonyl fluoride, was used to collect samples. Thereafter, 8% SDS- PAGE and polyvinylidene difluoride (PVDF) membranes were used to separate and transfer the protein, respectively. Blots were incubated with anti-AKT, anti-*p*-AKT, anti-ERK1/2, anti-*p*-ERK1/2, anti-Bcl-2, anti-pro-caspase 8, or anti- PARP-cleaved, respectively. Proteins were then washed three times with TBST for 5 min. Finally, the membranes were incubated with secondary antibodies for 1 h. The results were analyzed using an ECL detection system. GAPDH was used as the reference protein.

### Statistical Analysis

All experimental data were expressed as mean ± SD. Statistical analyses were performed using GraphPad Prism. Differences between groups were analyzed using Student’s unpaired *t*-test. IC50 values of ZnS in cell lines were calculated *via* nonlinear regression (curve fit). The 95 confidence intervals were calculated. Differences in the IC50 values of ZnS in the two cell lines were compared using the unpaired *t*-test. Statistically significance was set at *p* < 0.05.

## Results

### ZnS Inhibited the Proliferation of HCC Cells

The inhibitive effect of ZnS on HCC cell growth was studied by performing cell viability assays with different concentrations of ZnS (0–6.4 μM). The viability rate of the two HCC cell lines was inhibited by ZnS in a dose-dependent manner (Figure 1B), while the viability rate of normal cells remained stable. The IC50 value of ZnS in Huh7 cells was determined to be 0.51 μM, and that in SMMC-7721 cells was 1.0 μM. The IC50 values of ZnS in the two cell lines were significantly different. Thus, ZnS was able to effectively inhibit the growth of HCC cells but had less influence at low and medium concentrations.

### ZnS Promoted Apoptosis in HCC Cells

In order to determine the inhibitory effect of ZnS on HCC cells, we investigated the effect of ZnS on the apoptosis of HCC cells. Huh7 and SMMC-7721 cells were treated for 48 h with 0.51 μM ZnS and 1.0 μM ZnS, respectively. The cell apoptosis percentages of Huh7 and SMMC-7721 cells were increased significantly by ZnS treatment compared with those of the blank controls (Figures 1C,D). In addition, we found that the apoptosis percentage of the Huh7 cells was increased by DMSO treatment compared with blank control. These results suggested that the administration of ZnS to HCC cells significantly increased cell apoptosis. Because of the stronger inhibitive effect of ZnS in SMMC-7721, SMMC-7721 cell line was selected for further sequencing analysis.

### LncRNA Profiling and Validation

**FIGURE 2:**
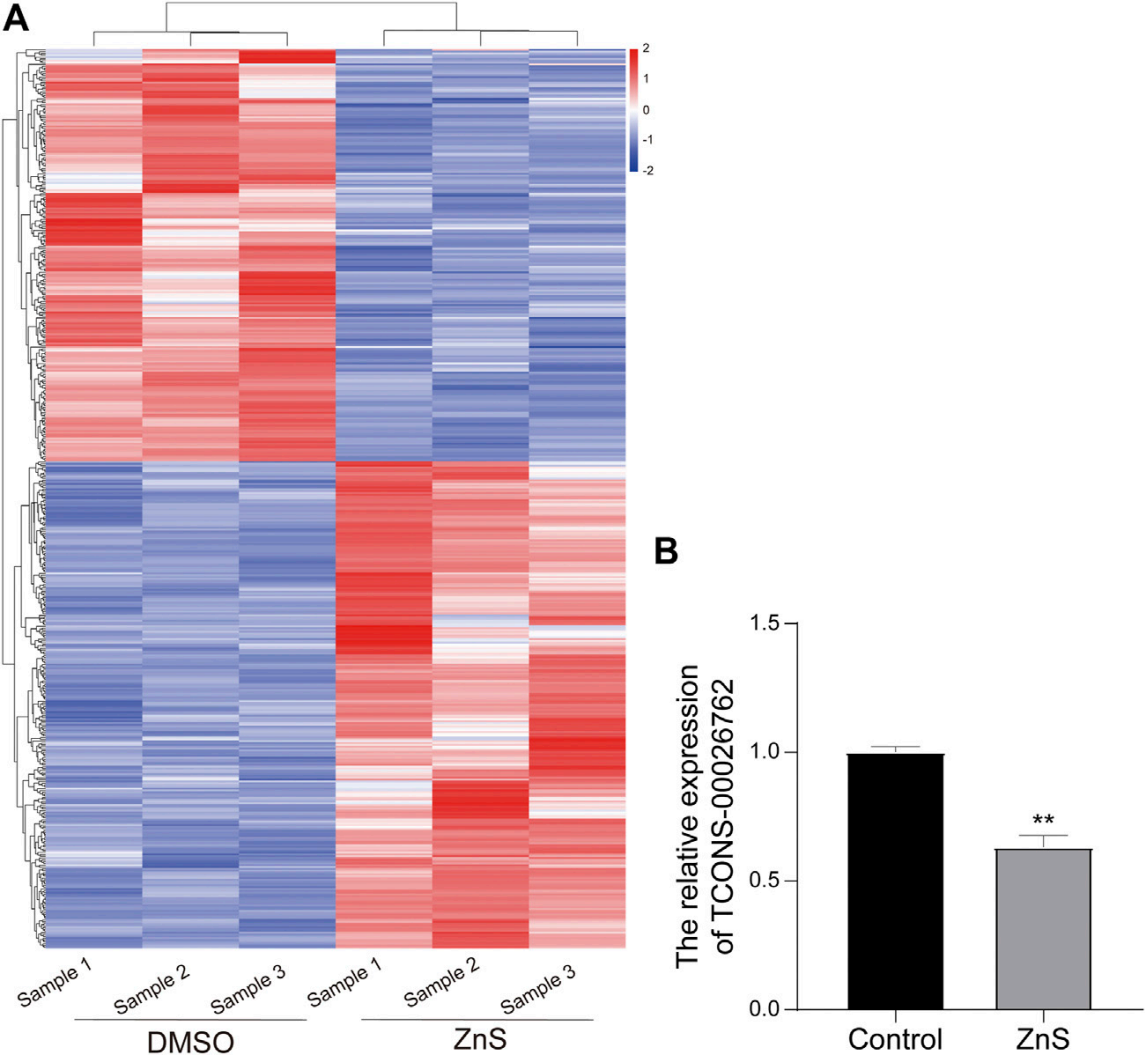
Analysis of differentially expressed lncRNAs treated with ZnS. **(A)** The heatmap of differentially expressed lncRNAs between DMSO and ZnS groups. **(B)** The qRT-PCR analysis of TCONS-00026,762 expression. Each trial was replicated at least three times. Data are expressed as mean ± SD, **, *p* < 0.01.

SMMC-7721 cells were treated with 1.0 μM ZnS or DMSO for 48 h before total cellular RNA was isolation. High-throughput sequencing was performed to detect lncRNAs. A total of 493 DElncRNAs, including 267 up-regulated and 226 down-regulated lncRNAs, were identified as differentially expressed lncRNAs in ZnS-treated SMMC-7721 cells (Figure 2A). To find lncRNA that might be related to the development and progression of hepatocellular carcinoma, we confirmed the location of top 30 differentially expressed lncRNAs and predicted their adjacent/localized genes through Ensemble BLAST. The results showed that the TCONS-00026762 is located on chromosome 10p15.1 and largely overlaps aldo-keto reductase family 1 member C1 (AKR1C1). AKR1C1 is reportedly involved in the initiation and progression of cancer (Chang et al., 2019; Huebbers et al., 2019; Wang et al., 2019; Zhao et al., 2019). So, lncRNA TCONS-00026762 was selected for further investigation. The result of qRT-PCR showed that the expression of TCONS-00026762 was markedly down-regulated in the ZnS group (Figure 2B).

### TCONS-00026762 Regulated the Movability of HCC Cells

**FIGURE 3:**
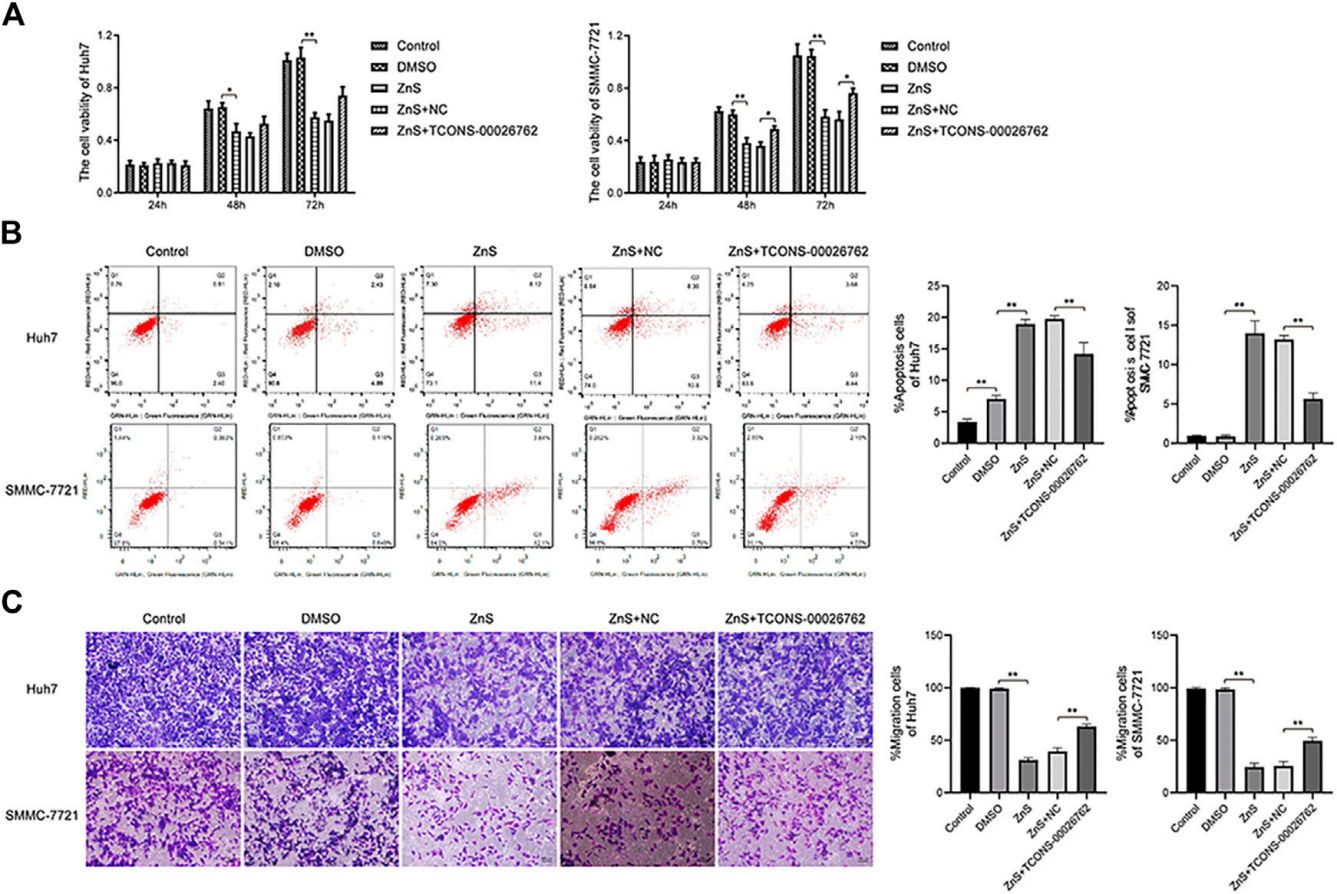
TCONS-00026,762 regulated the movability of HCC cells. **(A)** The viability rates of Huh7 and SMMC-7721 cells. **(B)** The flow cytometry analysis results and apoptosis rates of Huh7 and SMMC-7721 cells. **(C)** The invasion of Huh7 and SMMC-7721 cells were detected by transwell. Scale bar, 100 μm. Each trial was replicated at least three times. Data are expressed as mean ± SD, *, *p* < 0.05, **, *p* < 0.01.

TCONS-00026762 over-expression was established in Hun-7 and SMMC-7721 cell lines *via* lentiviral infection. ZnS significantly inhibited the proliferation of HCC cells. However, TCONS-00026762 could enhance the viability of HCC cells (Figure 3A). The flow cytometry analysis results were consistent with the above findings. ZnS treatment significantly induce apoptosis in HCC cells than in the blank control. TCONS-00026762 protected HCC cells from apoptosis (Figure 3B). Furthermore, transwell assays showed that ZnS significantly inhibited HCC cell invasion, while TCONS-00026762 promoted HCC cell invasion (Figure 3C).

### TCONS-00026762 Regulated SMMC-7721-Derived Tumor Progression

**FIGURE 4:**
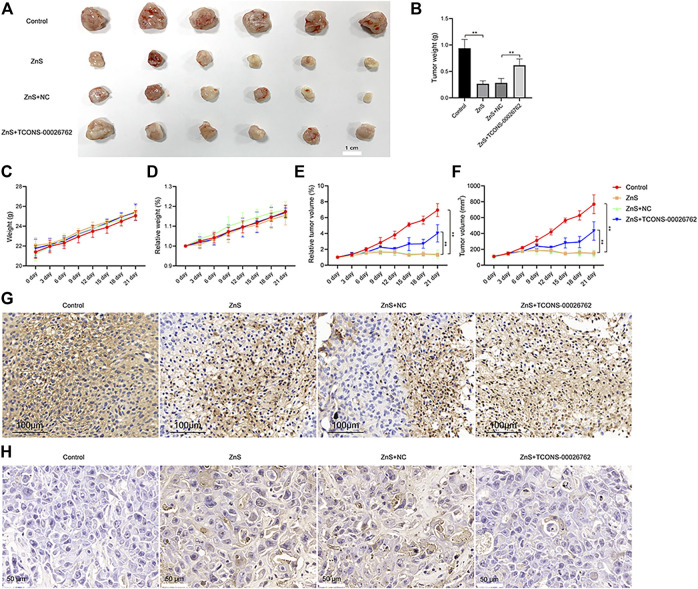
The inhibitory effect of ZnS and TCONS-00026762. **(A)** SMMC-7721 cells were subcutaneously injected into the right flank of nude mice to establish tumors. Scale bar, 1 cm. **(B)** The tumor weight measured **(C and D)** The weight and relative weight of nude mice were measured **(E and F)** The tumor volume and tumor relative weight were measured **(G)** Immunohistochemical detection of ki67 expression in tumor tissue. Scale bar, 100 μm **(H)** The apoptosis of tumor tissue was analyzed by TUNNEL. Scale bar, 50 μm. Error bars represents mean ± SD, **, *p* < 0.01.

We constructed a xenograft model by subcutaneously implanting SMMC-7721 cells in nude mice. Following 28 days of culturing, ZnS significantly inhibited tumor growth, while TCONS-00026762 caused significantly tumor formation (Figures 4A–F).

Ki67 protein is a widely used cellular marker of proliferation present during all active phases of the cell cycle (Zhao et al., 2017). We examined the expression of ki67 in tumors *via* immunohistochemistry (Figure 4G). The results indicated that ZnS suppressed the expression of ki67, whereas TCONS-00026762 improved ki67 expression. Furthermore, we detected cell apoptosis in tumors *via* TUNNEL. The results showed that ZnS induced apoptosis in tumor tissues, whereas TCONS-00026762 could reduce apoptosis (Figure 4H). The results above suggested that ZnS induced apoptosis in SMMC-7721-derived tumor progression by regulating the expression of TCONS-00026762.

### TCONS-00026762 Regulated the Expression of Apoptosis-Related Proteins

**FIGURE 5:**
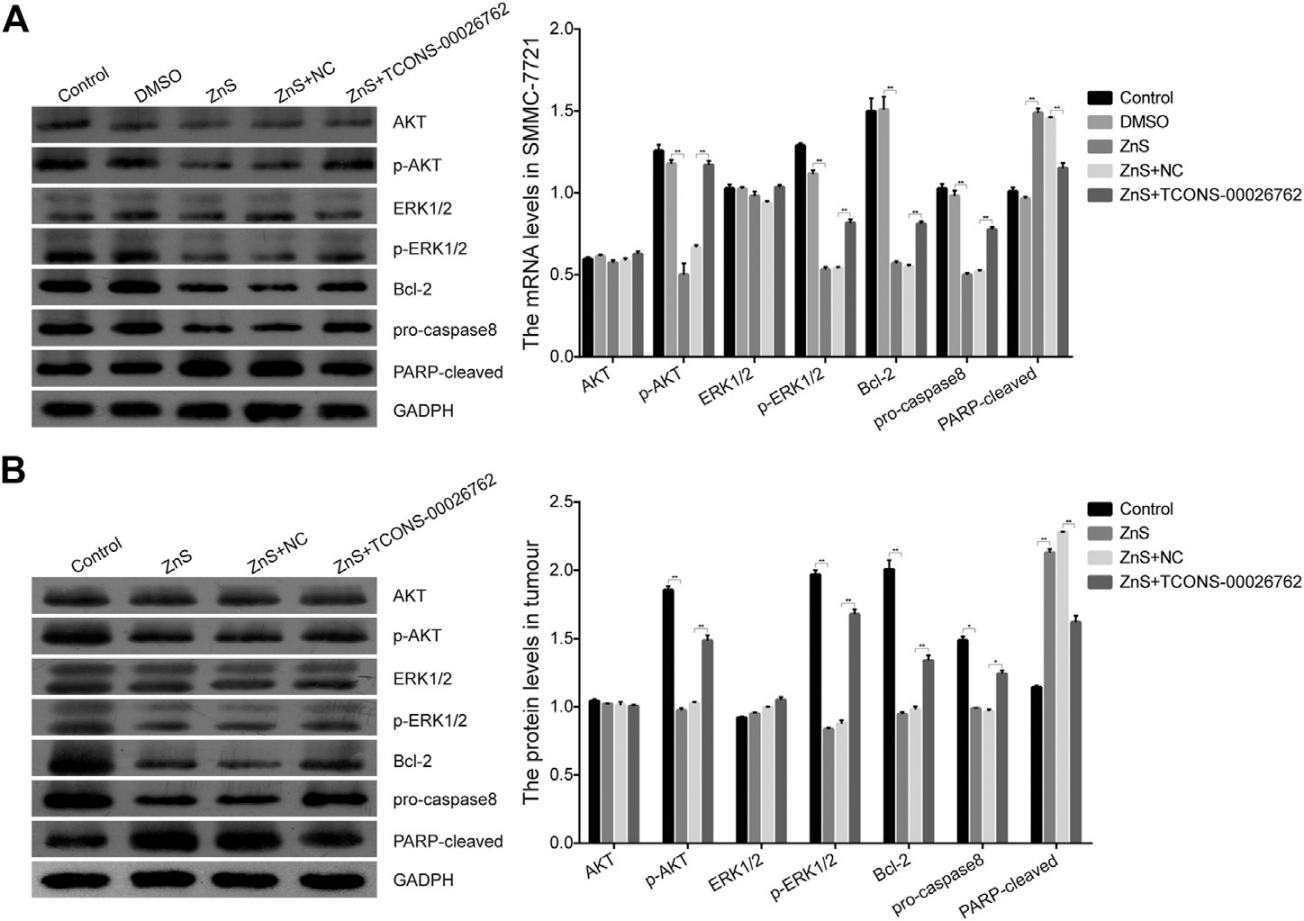
The expression levels of apoptosis-related proteins in SMMC-7721 cells **(A)** and tumor tissue **(B)**. Each treatment was replicated three times. Error bars represents mean ± SD, *, *p* < 0.05, **, *p* < 0.01.

Based on above results, we examined the expression of apoptosis-related proteins *in vitro* and *in vivo*. The results of the western blotting assay showed that the expressions of *p*-AKT, *p*-ERK1/2, Bcl-2 and pro-caspase8 were down-regulated in cells and tumor tissues treated with ZnS, while the expression of PARP-cleaved was up-regulated (Figure 5). In addition, TCONS-00026762 could up-regulated the phosphorylation of AKT and ERK1/2 and the expression of Bcl-2 and caspase8, and down-regulated the expression of PARP-cleaved (Figure 5). Overall, these results indicate that ZnS regulated the expression of apoptosis-related proteins by reducing the expression of TCONS-00026,762, there by inducing cell apoptosis.

## Discussion

HCC, which is widely considered as one of the most aggressive and lethal malignant tumors, is a great threat to human health (Ferlay et al., 2015; Bray et al., 2018). Therefore, it is crucial to identify effective drugs or treatments that can reduce the risk of metastasis and recurrence of HCC. Traditional Chinese medicines or natural extracts, which display high levels of efficacy and low toxicity compared to other drugs, have shown much promise in this direction (Karimi et al., 2013; Giordani et al., 2015). ZnS is used in the treatment of various cancers and therefore may be an effective drug against HCC. We investigated the inhibitory effect of ZnS on HCC as well as the regulation mechanisms underlying lncRNA expression.

Reportedly, ZnS exerts cytotoxic activity against many cancers, such as colon carcinoma, lung carcinoma and melanoma (Tong et al., 2012). ZnS inhibited proliferation and induced apoptosis in C26 cells (colon carcinoma), HepG2 (HCC), A549 (lung carcinoma) and B16 (melanoma) (Tong et al., 2012). Zhao et al., reported that the zingiberenesis saponin I increased the expression levels of cleaved PARP, caspase-3/9, and Bax, and reduced Bcl-2 in C26 cells. Administering zingiberenesis saponin I promoted DNA fragmentation in C26 cells was (Qingbing et al., 2010). Our present study suggested that administering ZnS to Huh7 and SMMC-7721 cells promoted cell apoptosis and inhibited proliferation in a dose-dependent manner. These results suggested that ZnS exerts an antitumor effect on HCC cells by inhibiting cell proliferation and promoting apoptosis.

The anticancer mechanism of ZnS may be closely related to lncRNA. It has been reported that ingredients of D. zingiberensis Wright, such as saponin and dioscin, inhibited the growth of gastric cancer cells (HGC-27, MGC803 and SGC7901) by decreasing the expression of lncRNA HOTAIR (Ma et al., 2016). Our Illumina sequencing analysis showed that ZnS induced dysregulation of lncRNA in SMMC-7721 cells. These results suggested that these DElncRNAs play important roles in ZnS-mediated antitumor effects in HCC cells. The novel lncRNA TCONS-00026762 attracted our attention due to its largely unknown role in HCC progression. Blast in NCBI, TCONS-00026762 largely overlaps with the promoter and exon of AKR1C1. AKR1C1 is a member of the human aldo-keto reductase family, which catalyze NADPH-dependent reductions (Dhagat et al., 2008; Rizner and Penning, 2014; Zhu et al., 2018). Mounting evidence reveals that AKR1C1 is volved in a variety of cancers, including hepatocellular carcinoma, lung cancer, gastric cancer, and cervical cancer, and the over-expression of AKR1C1 has been found to be associated with carcinogenesis (Hsu et al., 2001; Ji et al., 2004; Fukumoto et al., 2005; Woenckhaus et al., 2006; Seo et al., 2007; Chang et al., 2009; Chien et al., 2009; Rizner, 2012; Matsunaga et al., 2014). In addition, the low expression of AKR1C1 may slow down the progression of cancer (Klippel et al., 2012). All these results suggest that AKR1C1 plays an important role in carcinogenesis. Therefore, TCONS-00026762 was selected for further experiments.

In order to investigate the function of TCONS-00026762 in HCC progression, TCONS-00026762 was over-expressed in the SMMC-7721 cell line *via* lentivirus packaging. The results showed that TCONS-00026762 could reduce the effect of ZnS on HCC cells, suggesting that ZnS inhibits cell proliferation and invasion and induces cell apoptosis by suppressing the expression of TCONS-00026762. To determine the effect of TCONS-00026762 on tumorigenesis *in vivo*, we constructed mouse transplanted tumor models, using in nude mice transplanted with SMMC-7721 cells. ZnS dramatically suppressed the tumor growth and promoted cell apoptosis, as expected. Furthermore, ZnS significantly inhibited the effect of TCONS-00026762 on tumor progression.

Cell apoptosis is a form of programmed cell death, which is regulated by multiple genes and it is an effective cellular mechanism against cancer (Renault and Chipuk, 2014; Song et al., 2018). According to a previous study, PI3K/AKT signaling pathway plays an important role in cell apoptosis. Inhibition of PI3K/Akt signaling pathway could inhibit tumor growth and promote apoptosis (Fang et al., 2007; Zhou et al., 2020). ERK1 and ERK2, collectively referred to as ERK 1/2, regulate multiple biological processes including development, apoptosis, and inflammation. (Joshi, 2014). Bcl-2, an apoptosis inhibitory protein, play an important role in the regulation of apoptosis. Inhibition of Bcl-2 promoted the apoptosis (Shimizu et al., 1999). Pro-caspase8 is reportedly activated by autocatalytic cleavage, and activated caspase-8 induces cell death (Muzio et al., 1998). PARP is a key enzyme in the detection of DNA damage and occurrence. PARP is cleaved to 89-kDa fragment form, which could induce apoptosis through the access of endonucleases to chromatin. In the present study, we observed that ZnS significantly inhibit the activation of the *p*-Akt, *p*-ERK, Bcl-2 and pro-caspase8 and up-regulated expression and cleavage of PARP. And TCONS-00026762 offset the effects of ZnS, indicating that ZnS promotes the apoptosis of HCC cells by suppressing the expression of TCONS-00026762.

This study was beset by some limitations. TCONS-00026762 largely overlaps with AKR1C1, indicating a possible association between TCONS-00026762 and AKR1C1. This relationship was not validated in this study. Therefore, the combined effect of TCONS-00026762 and AKR1C1 on HCC progression requires further investigation. Additionally, due to the enhanced tissue specificity of lncRNAs and the ability of lncRNAs to function as competing endogenous RNAs (ceRNAs) to regulate microRNA/mRNA, therapeutic targeting of lncRNAs may result in fewer off-target effects than other therapeutic strategies (Ballantyne et al., 2016). These characteristics highlight the idea that therapeutic approaches that target lncRNAs may be faster and more efficient than methods that target proteins. Accordingly, we suggest that lncRNAs could be utilized as effective therapeutic targets for HCC in future.

In conclusion, ZnS, as a traditional Chinese medicine, not only inhibited the growth and invasion capability of HCC cells, but also promoted the apoptosis of HCC cells and tumors by suppressing lncRNA TCONS-00026762 expression. These findings suggest that ZnS could be an effective drug for the treatment of HCC in the future.

## Data Availability

The data presented in the study are deposited in the BioProject database, accession number: PRJNA718909.
