# Supplementary figures and images for: Dioscorea Zingiberensis New Saponin Inhibits the Growth of Hepatocellular Carcinoma by Suppressing the Expression of Long Non-coding RNA TCONS-00026762
Source: Front Pharmacol. 2021 May 3;12:678620. doi: 10.3389/fphar.2021.678620 (PMC8126712; doi:10.3389/fphar.2021.678620)

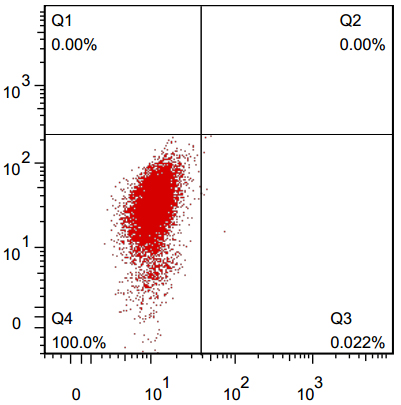

Supplement: Supplementary file 1 [file DataSheet1.ZIP › Original data/Figure 1/Figure 1C 1.jpg]

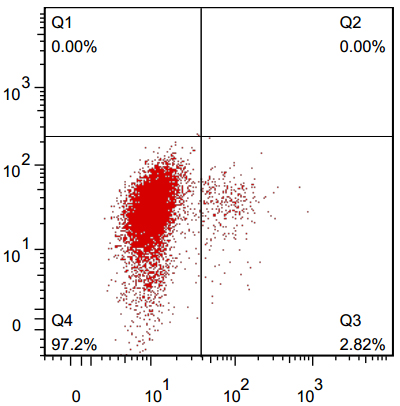

Supplement: Supplementary file 1 [file DataSheet1.ZIP › Original data/Figure 1/Figure 1C 2.jpg]

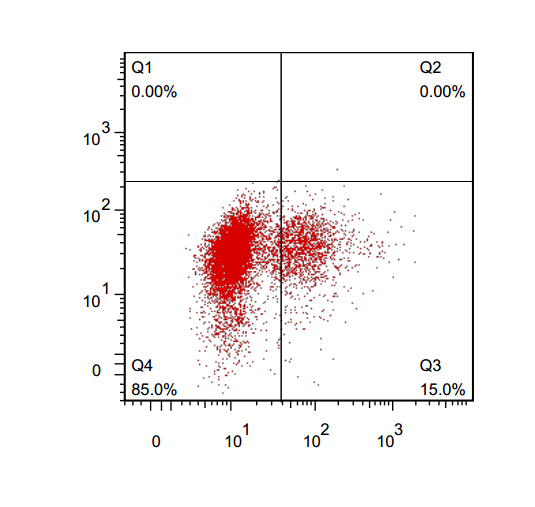

Supplement: Supplementary file 1 [file DataSheet1.ZIP › Original data/Figure 1/Figure 1C 3.jpg]

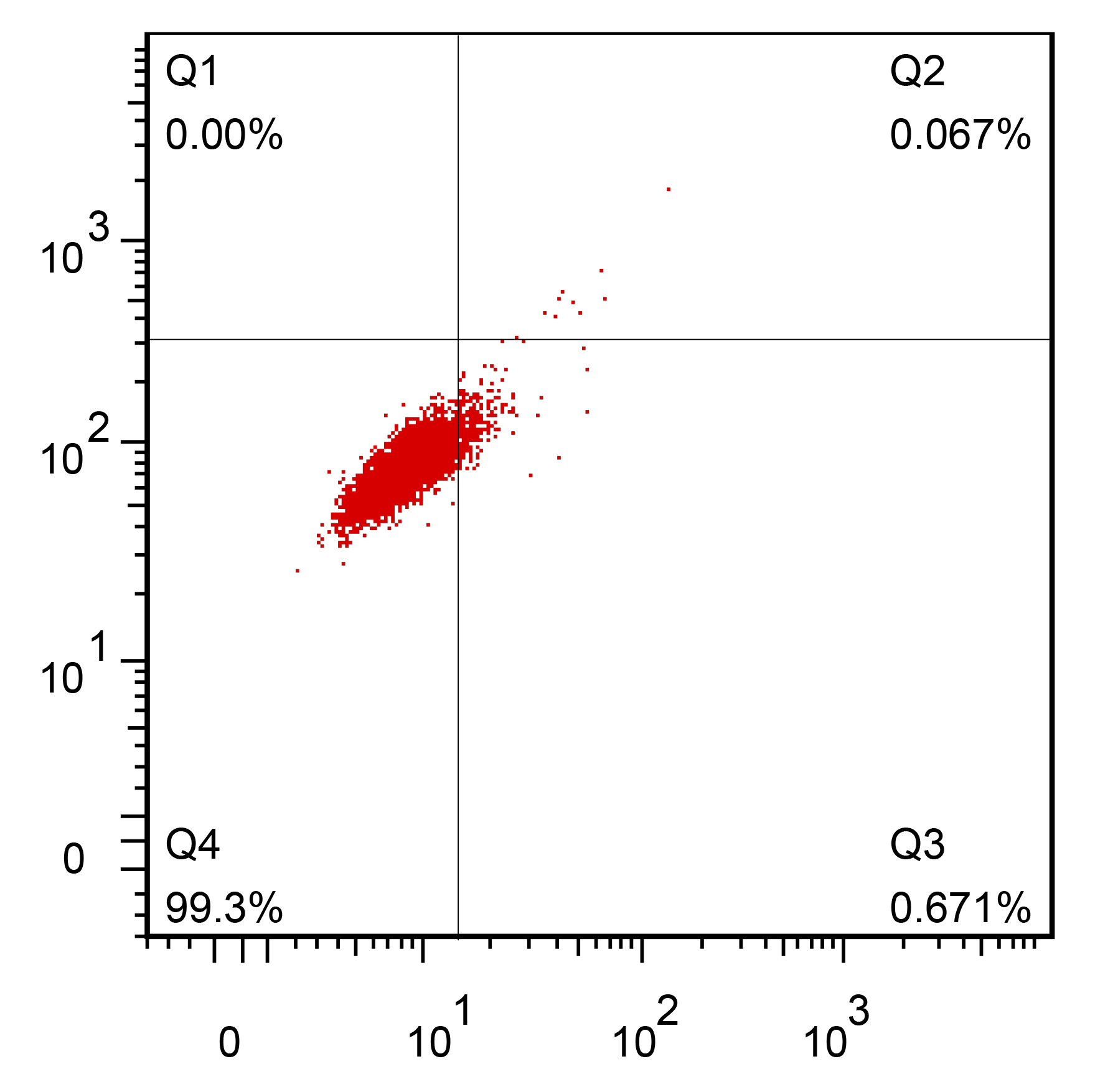

Supplement: Supplementary file 1 [file DataSheet1.ZIP › Original data/Figure 1/Figure 1D 1.jpg]

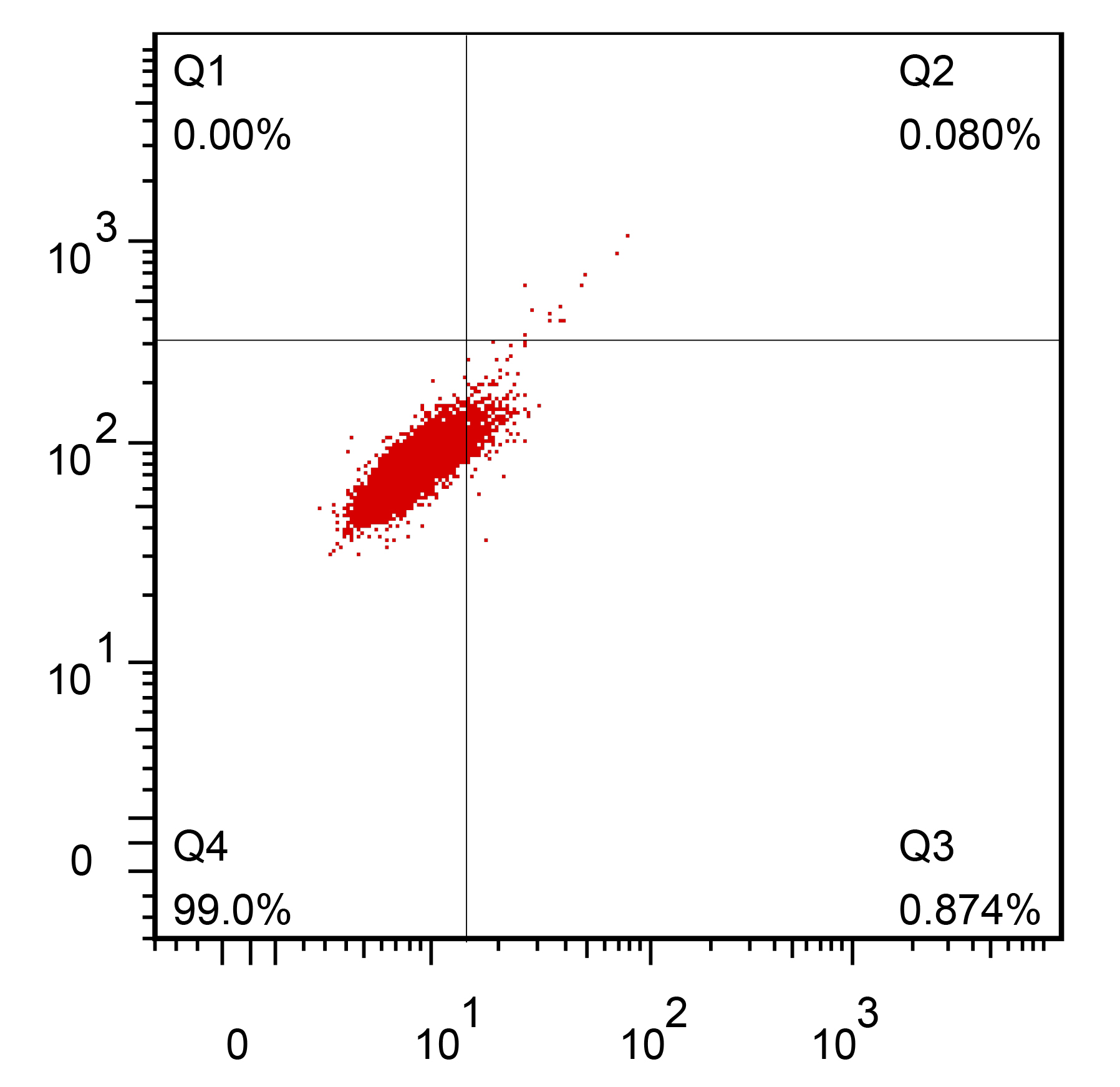

Supplement: Supplementary file 1 [file DataSheet1.ZIP › Original data/Figure 1/Figure 1D 2.jpg]

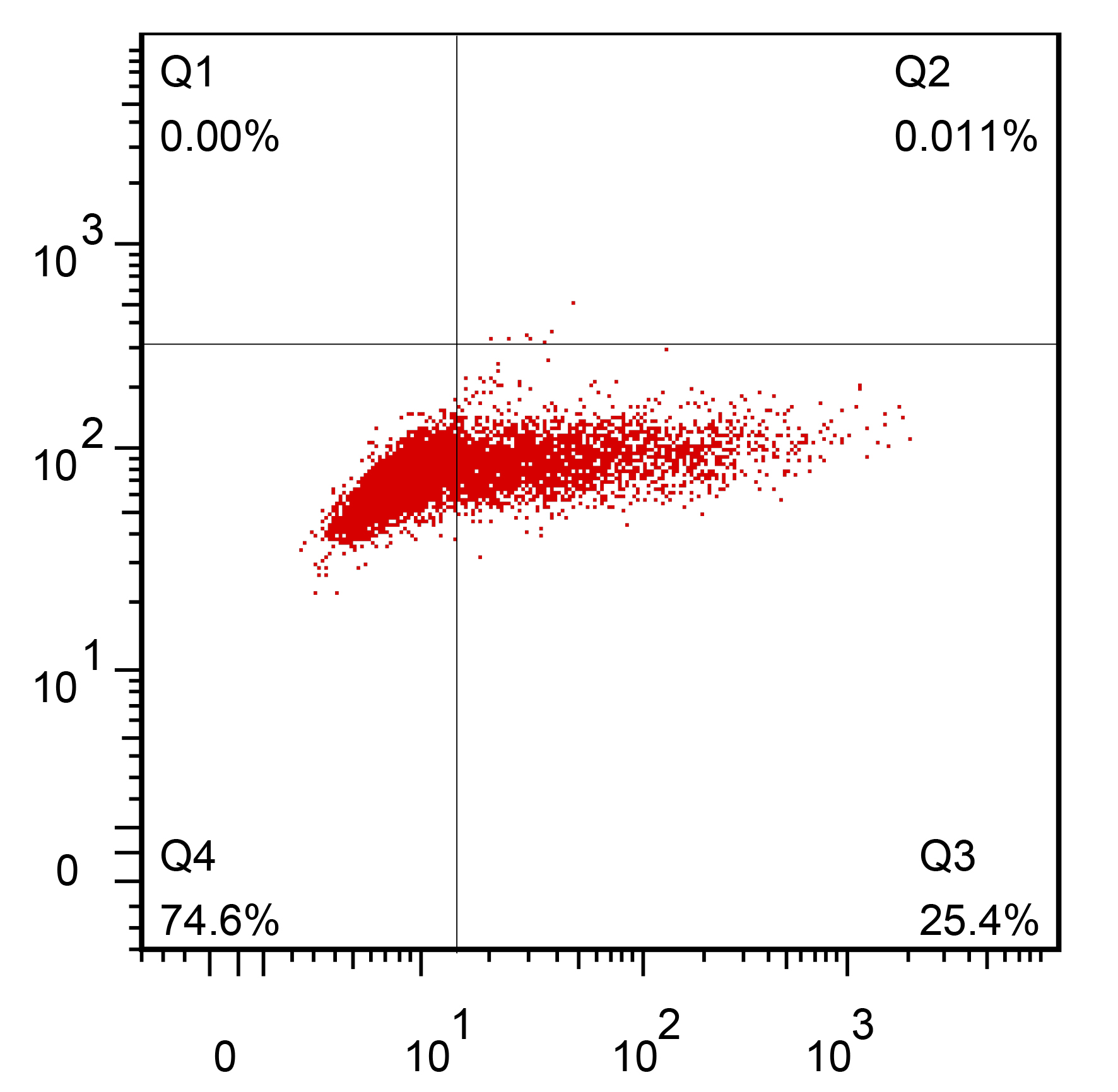

Supplement: Supplementary file 1 [file DataSheet1.ZIP › Original data/Figure 1/Figure 1D 3.jpg]

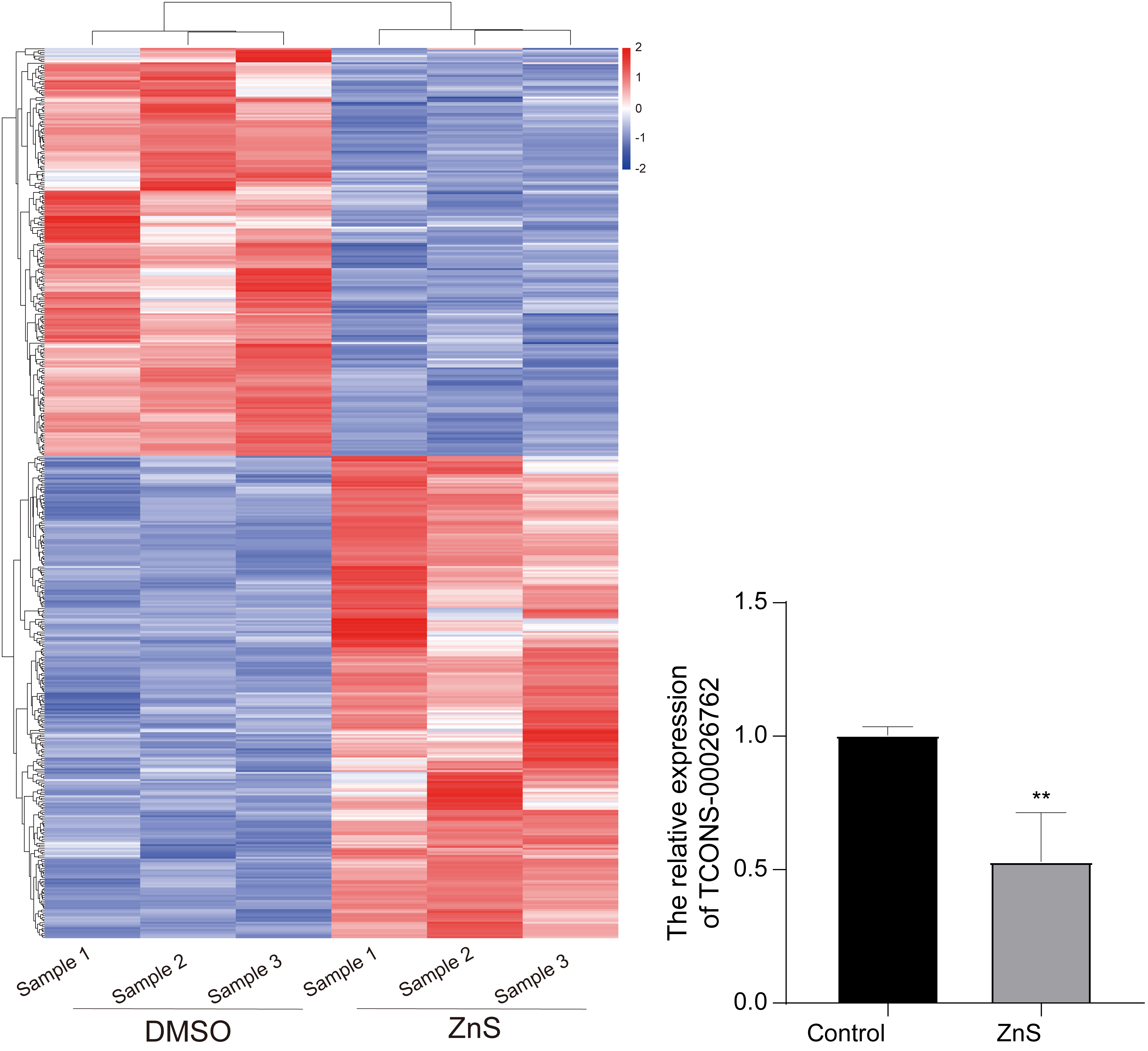

Supplement: Supplementary file 1 [file DataSheet1.ZIP › Original data/Figure 2/Figure 2.tif]

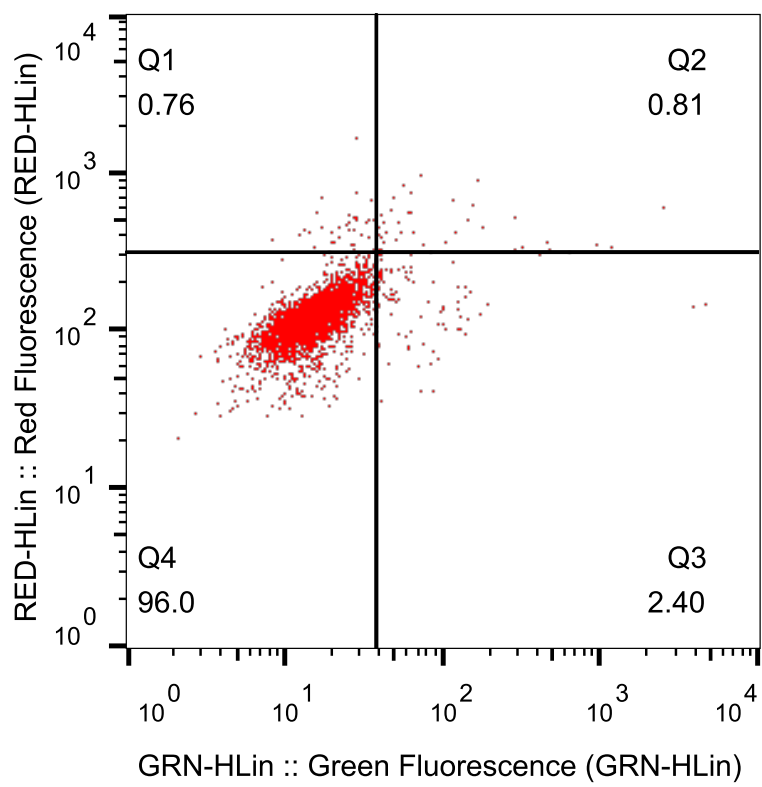

D:\LST\20210311\1.fcs

Lymphocytes

4091

Supplement: Supplementary file 1 [file DataSheet1.ZIP › Original data/Figure 3/Figure 3B Huh7 1.pdf]

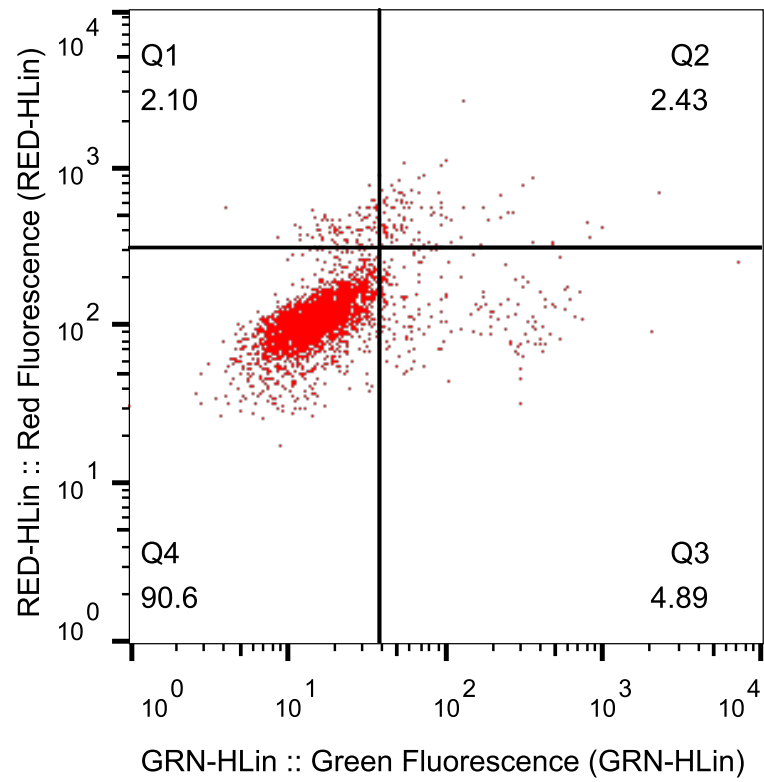

D:\LST\20210311\1.fcs

Lymphocytes

3909

Supplement: Supplementary file 1 [file DataSheet1.ZIP › Original data/Figure 3/Figure 3B Huh7 2.pdf]

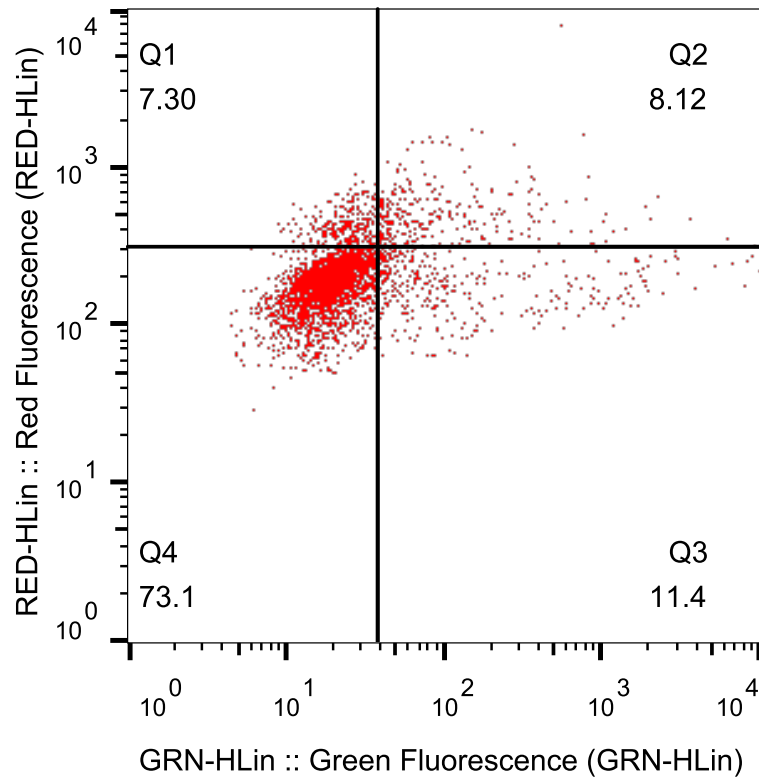

D:\LST\20210311\1.fcs

Lymphocytes

3385

Supplement: Supplementary file 1 [file DataSheet1.ZIP › Original data/Figure 3/Figure 3B Huh7 3.pdf]

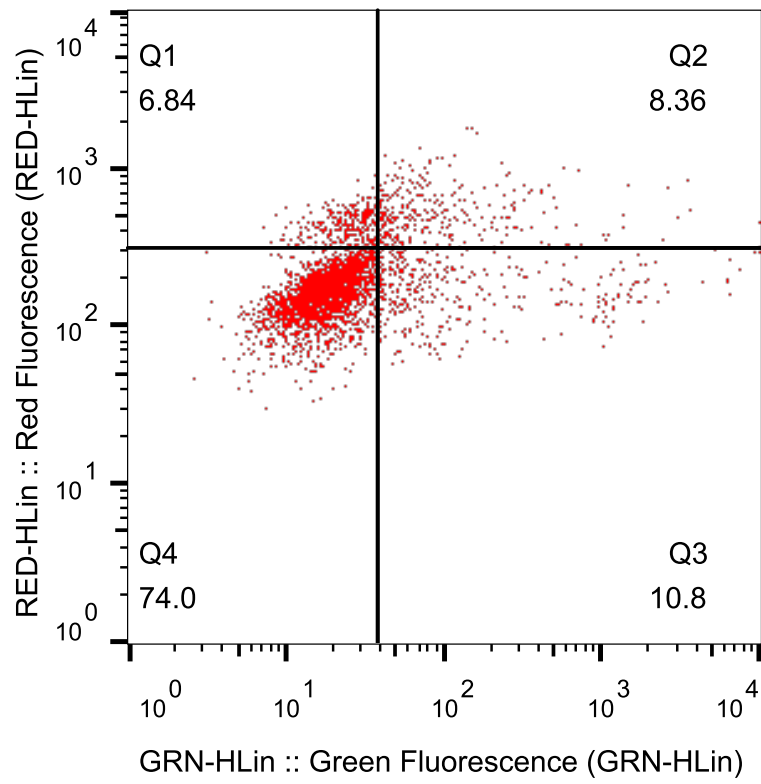

D:\LST\20210311\1.fcs  
Lymphocytes  
3479

Supplement: Supplementary file 1 [file DataSheet1.ZIP › Original data/Figure 3/Figure 3B Huh7 4.pdf]

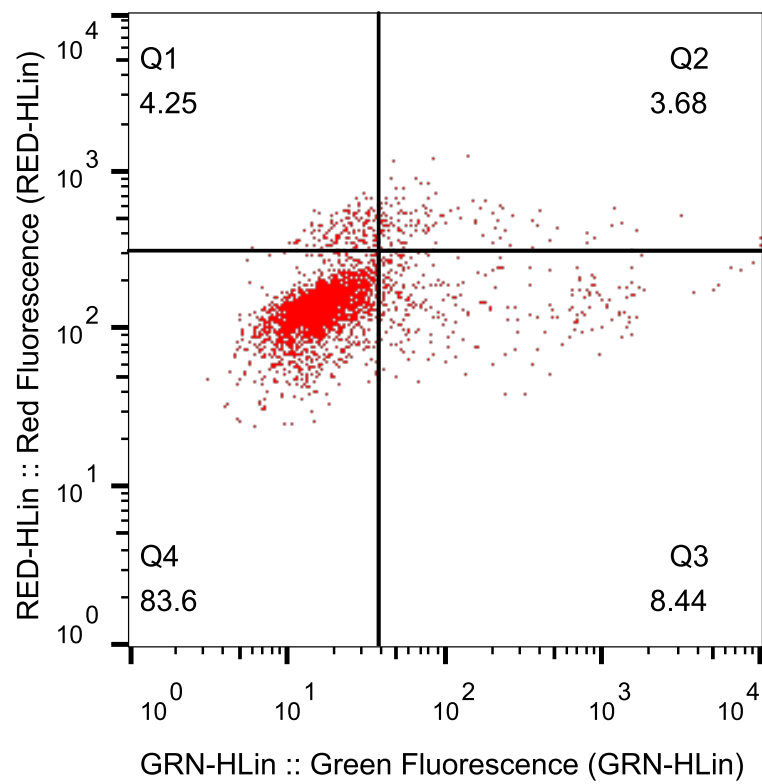

D:\LST\20210311\1.fcs  
Lymphocytes  
3365

Supplement: Supplementary file 1 [file DataSheet1.ZIP › Original data/Figure 3/Figure 3B Huh7 5.pdf]

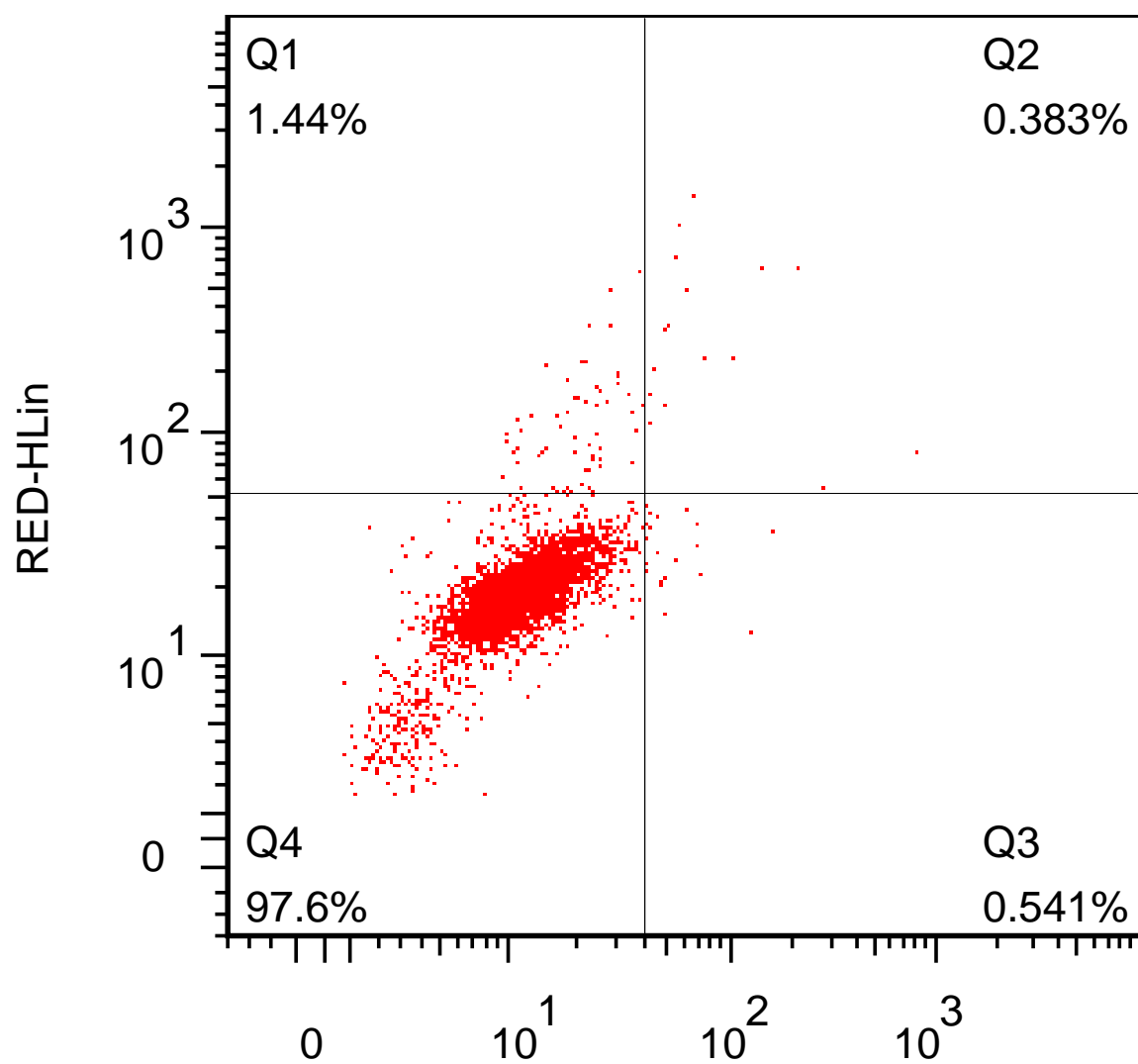

GRN-HLin:: Green Fluorescence (GRN-HLin)

1 (2).fcs

Forward Scatter (FSC-HLin), Side Scatter (SSC-HLin) s  
4435

Supplement: Supplementary file 1 [file DataSheet1.ZIP › Original data/Figure 3/Figure 3B SMMC-7721 1.pdf]

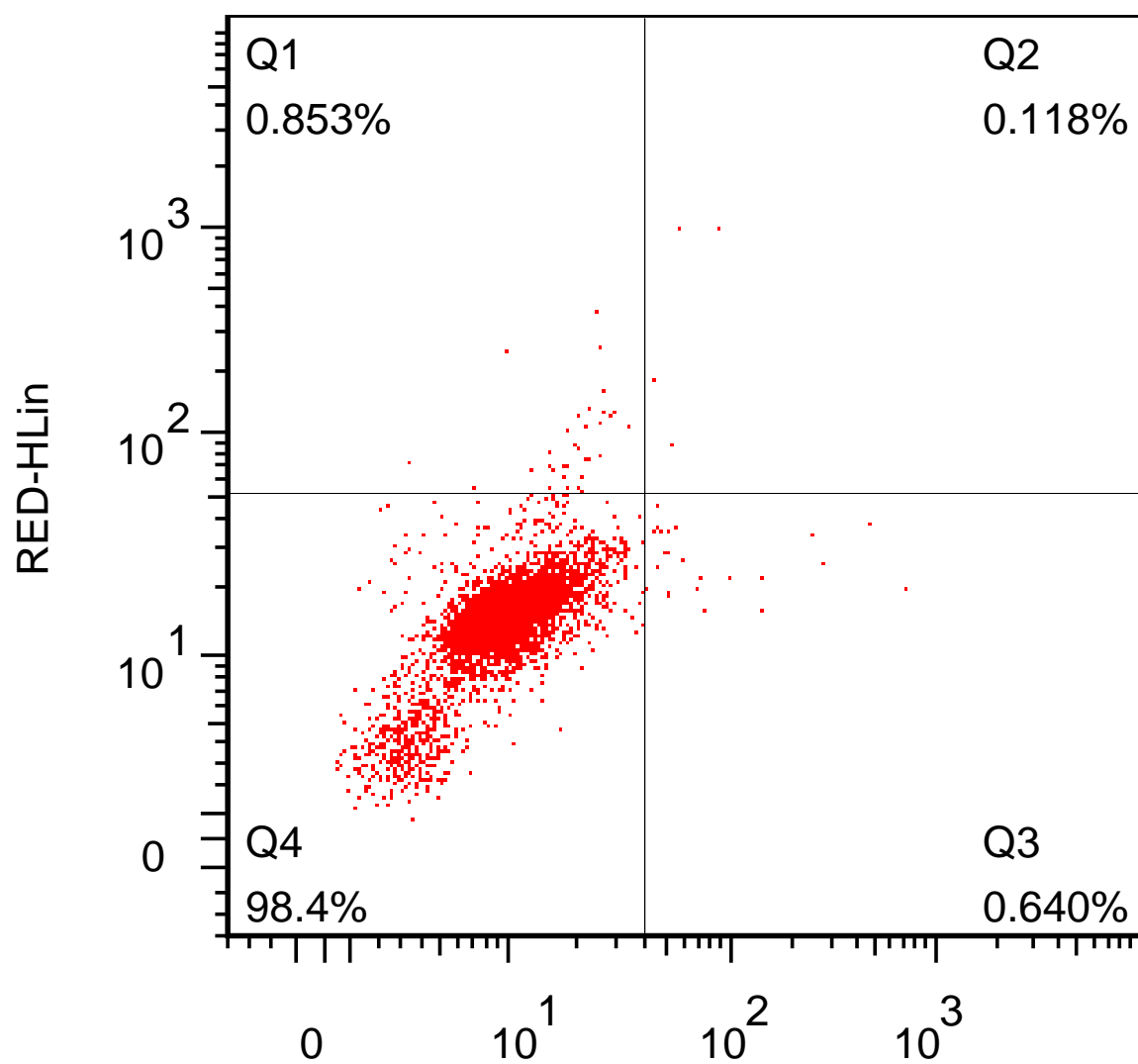

GRN-HLin:: Green Fluorescence (GRN-HLin)

1 (3).fcs

Forward Scatter (FSC-HLin), Side Scatter (SSC-HLin) s  
4221

Supplement: Supplementary file 1 [file DataSheet1.ZIP › Original data/Figure 3/Figure 3B SMMC-7721 2.pdf]

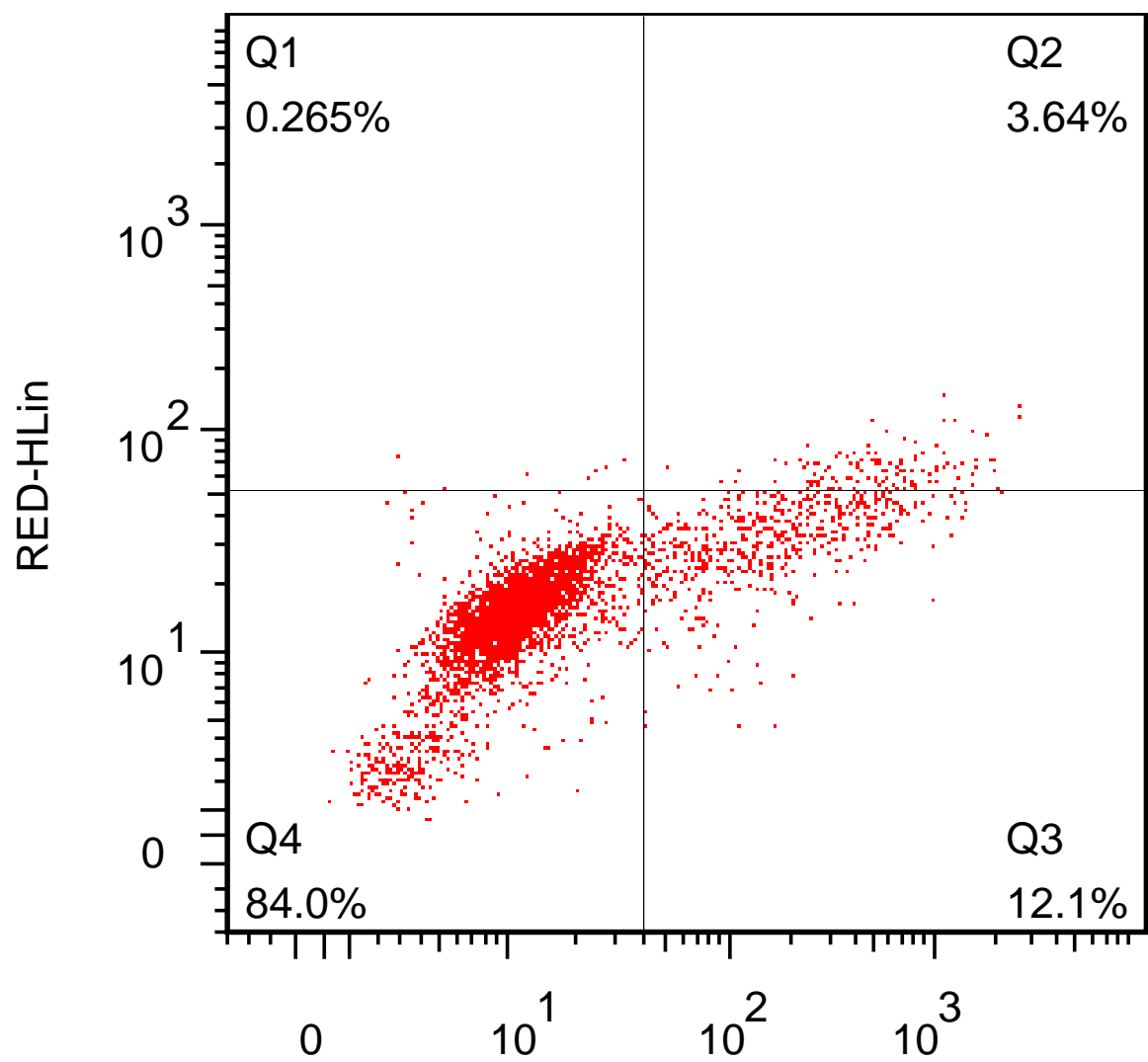

GRN-HLin:: Green Fluorescence (GRN-HLin)

1 (3).fcs

Forward Scatter (FSC-HLin), Side Scatter (SSC-HLin) s  
4147

Supplement: Supplementary file 1 [file DataSheet1.ZIP › Original data/Figure 3/Figure 3B SMMC-7721 3.pdf]

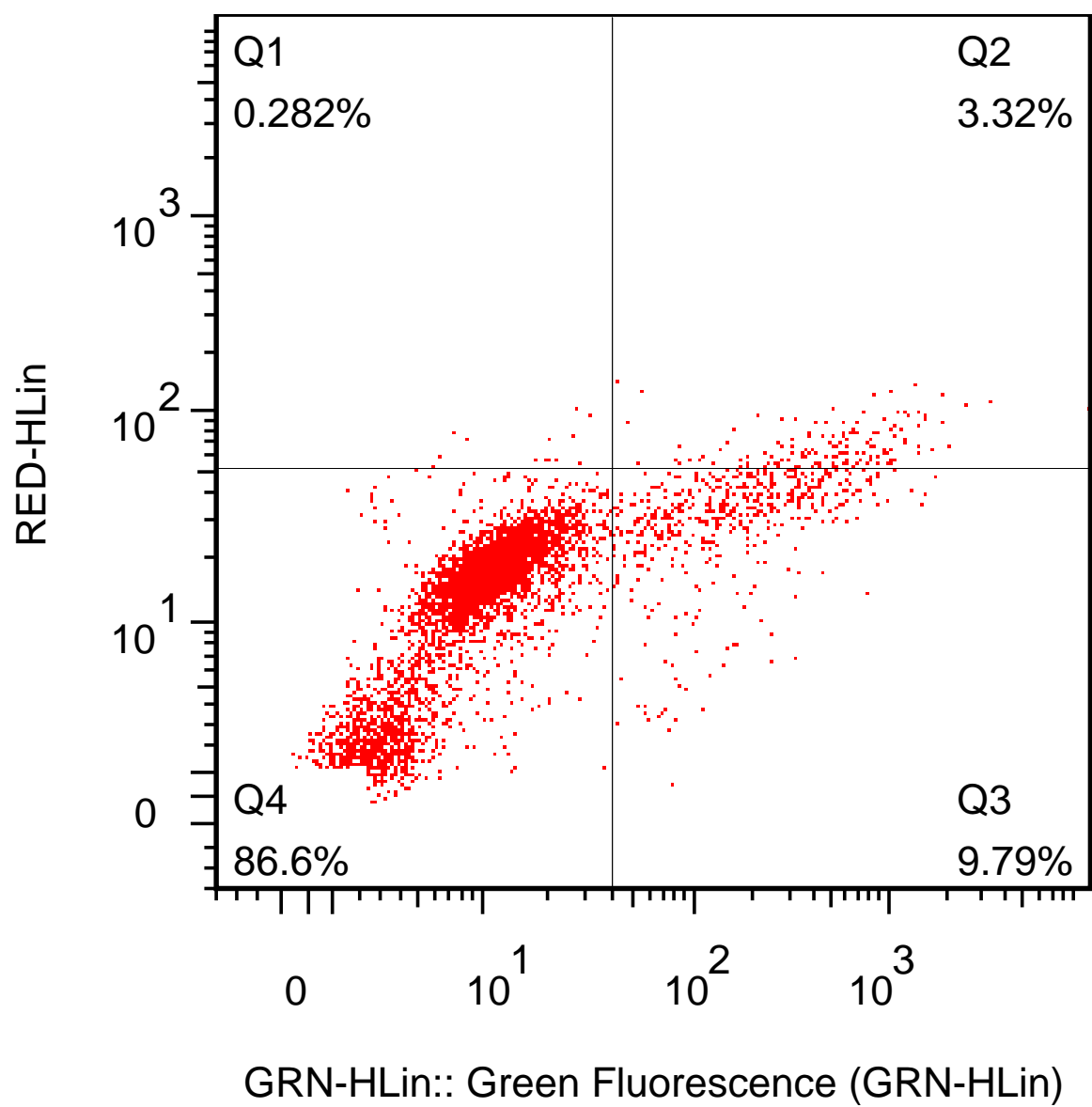

1 (2).fcs

Forward Scatter (FSC-HLin), Side Scatter (SSC-HLin) s  
4966

Supplement: Supplementary file 1 [file DataSheet1.ZIP › Original data/Figure 3/Figure 3B SMMC-7721 4.pdf]

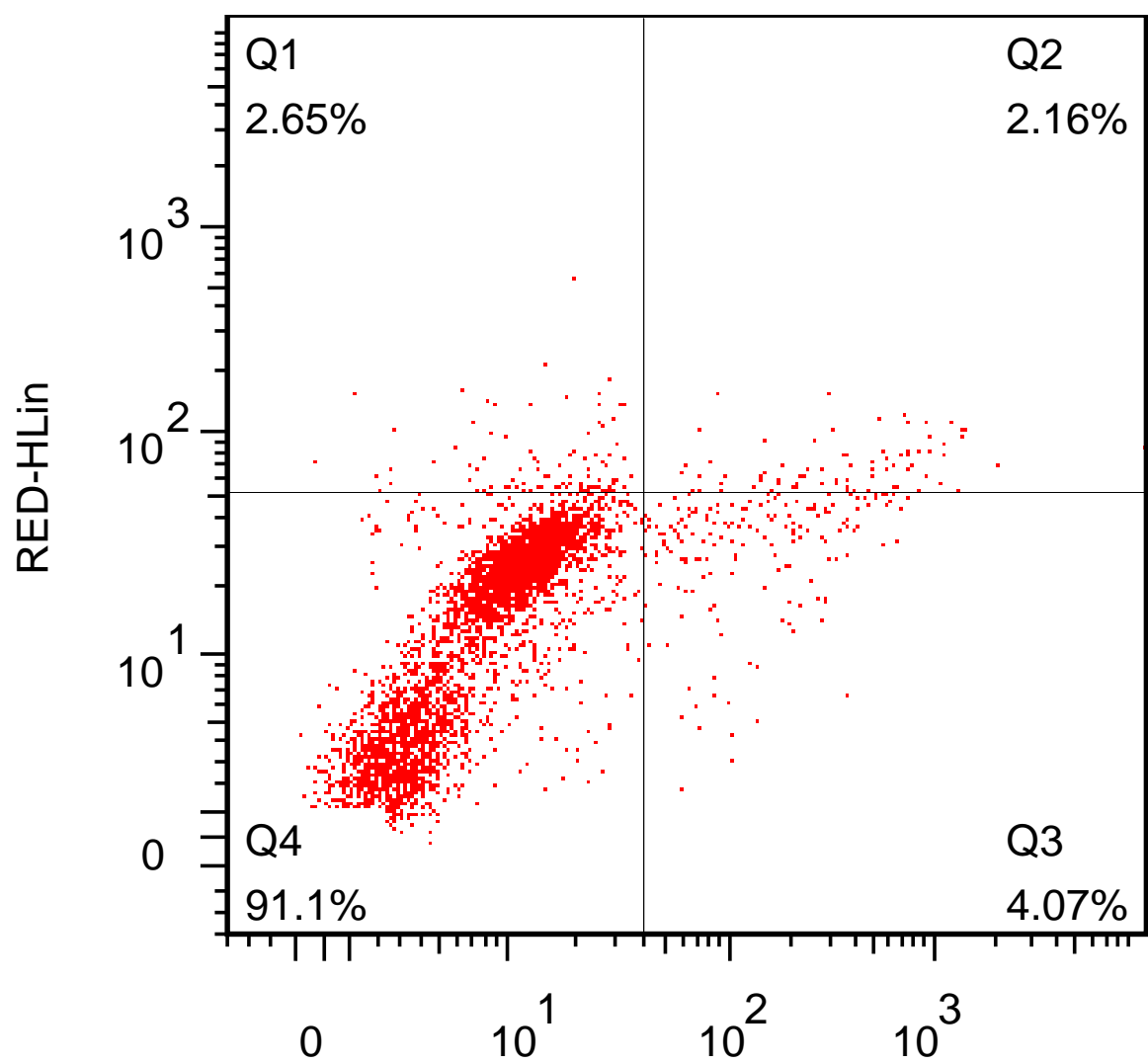

GRN-HLin:: Green Fluorescence (GRN-HLin)

1 (1).fcs

Forward Scatter (FSC-HLin), Side Scatter (SSC-HLin) s  
4033

Supplement: Supplementary file 1 [file DataSheet1.ZIP › Original data/Figure 3/Figure 3B SMMC-7721 5.pdf]

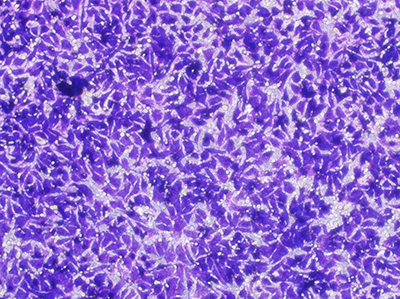

Supplement: Supplementary file 1 [file DataSheet1.ZIP › Original data/Figure 3/Figure 3C Huh7 1.tif]

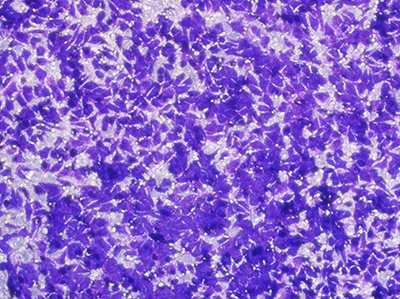

Supplement: Supplementary file 1 [file DataSheet1.ZIP › Original data/Figure 3/Figure 3C Huh7 2.tif]

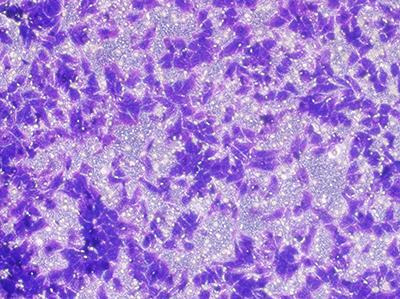

Supplement: Supplementary file 1 [file DataSheet1.ZIP › Original data/Figure 3/Figure 3C Huh7 3.tif]

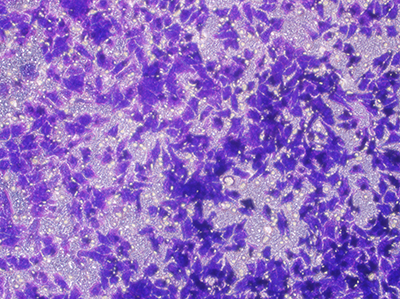

Supplement: Supplementary file 1 [file DataSheet1.ZIP › Original data/Figure 3/Figure 3C Huh7 4.tif]

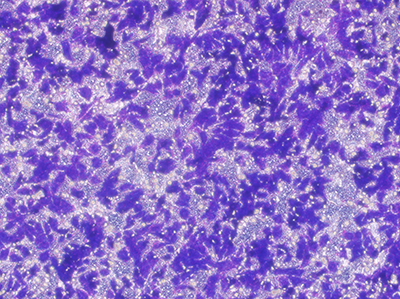

Supplement: Supplementary file 1 [file DataSheet1.ZIP › Original data/Figure 3/Figure 3C Huh7 5.tif]

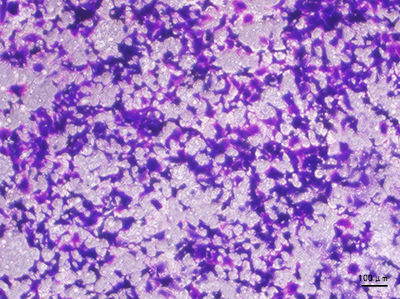

Supplement: Supplementary file 1 [file DataSheet1.ZIP › Original data/Figure 3/Figure 3C SMMC-7721 1.tif]

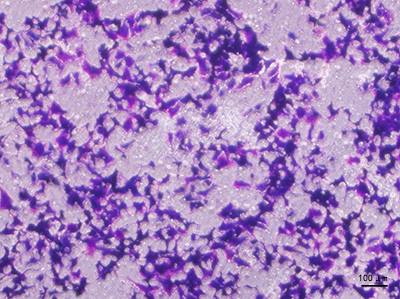

Supplement: Supplementary file 1 [file DataSheet1.ZIP › Original data/Figure 3/Figure 3C SMMC-7721 2.tif]

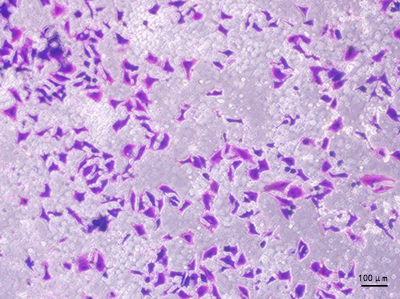

Supplement: Supplementary file 1 [file DataSheet1.ZIP › Original data/Figure 3/Figure 3C SMMC-7721 3.tif]

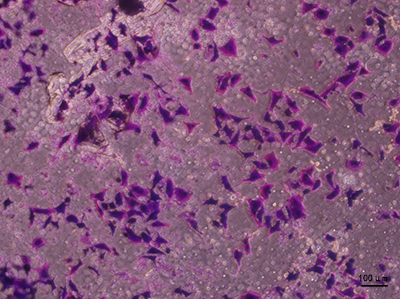

Supplement: Supplementary file 1 [file DataSheet1.ZIP › Original data/Figure 3/Figure 3C SMMC-7721 4.tif]

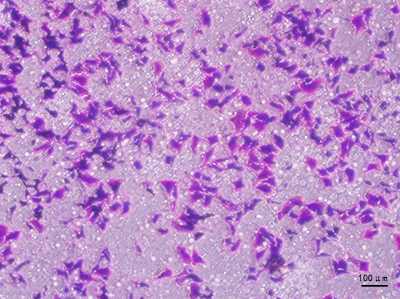

Supplement: Supplementary file 1 [file DataSheet1.ZIP › Original data/Figure 3/Figure 3C SMMC-7721 5.tif]

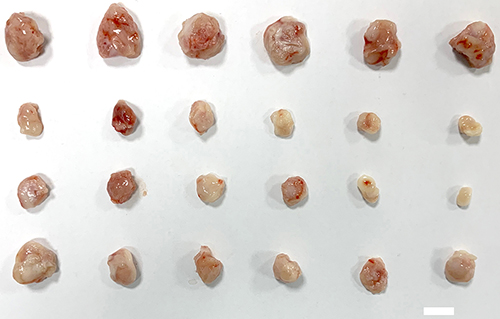

Supplement: Supplementary file 1 [file DataSheet1.ZIP › Original data/Figure 4/Figure 4A.jpg]

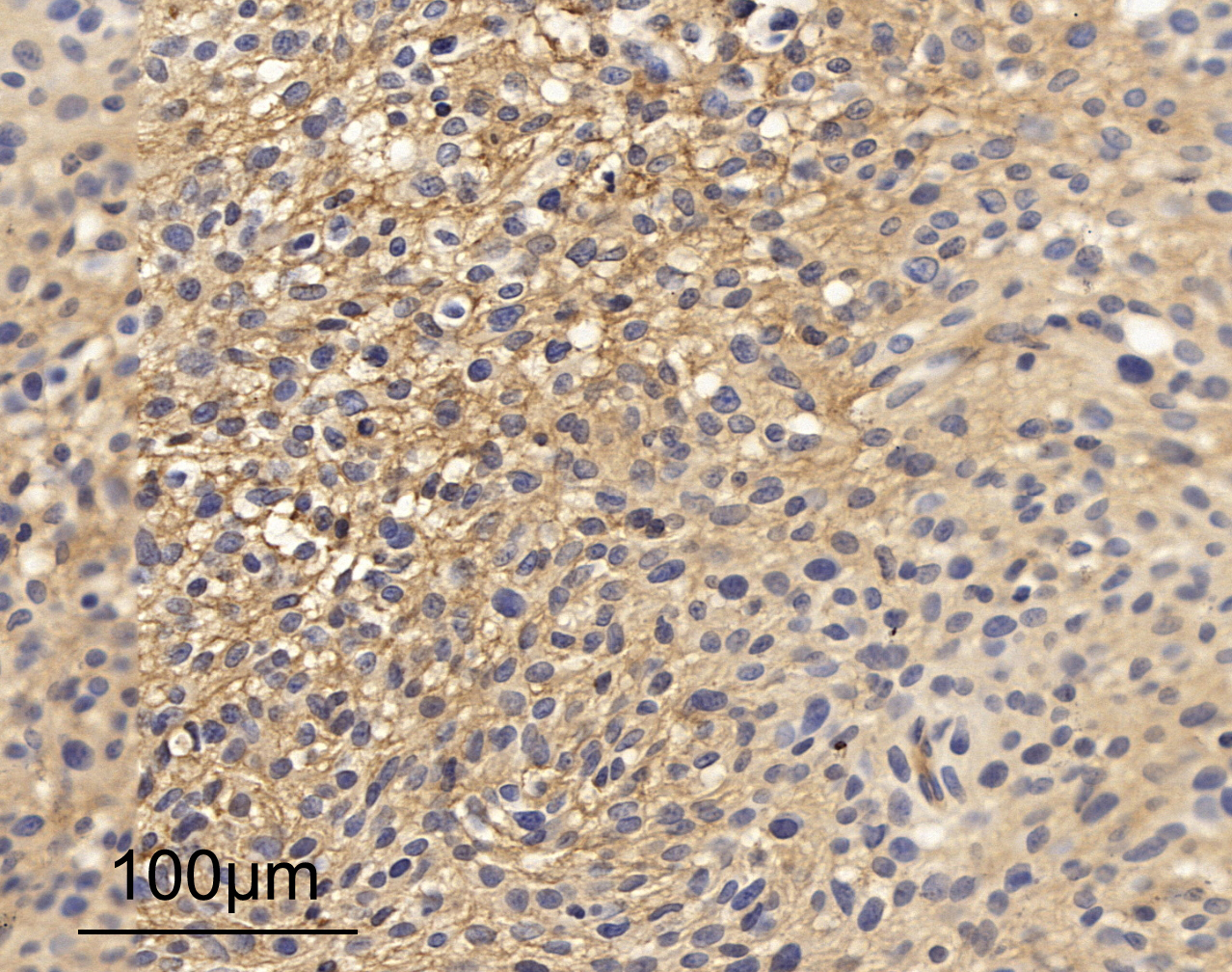

Supplement: Supplementary file 1 [file DataSheet1.ZIP › Original data/Figure 4/Figure 4G 1.jpg]

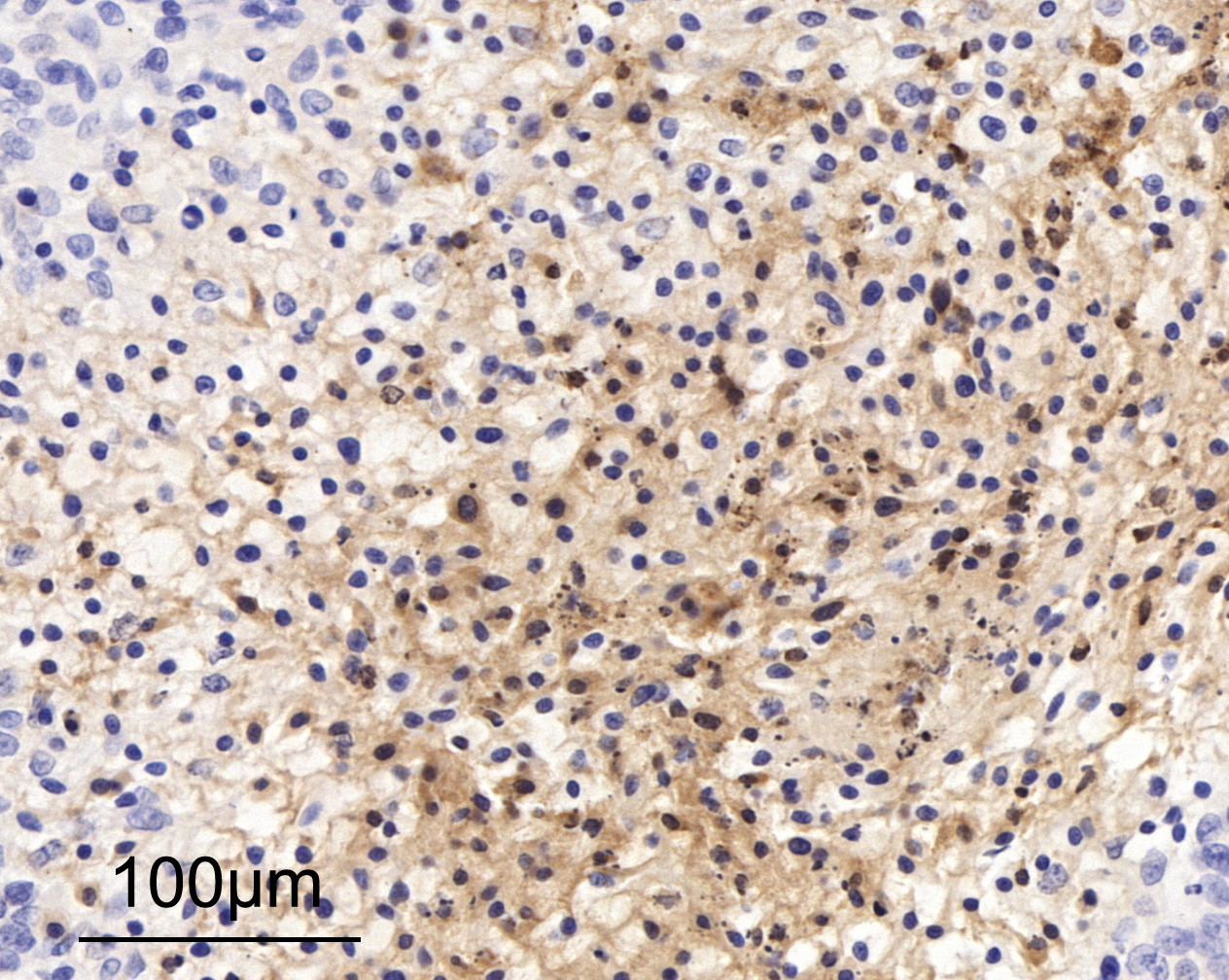

Supplement: Supplementary file 1 [file DataSheet1.ZIP › Original data/Figure 4/Figure 4G 2.jpg]

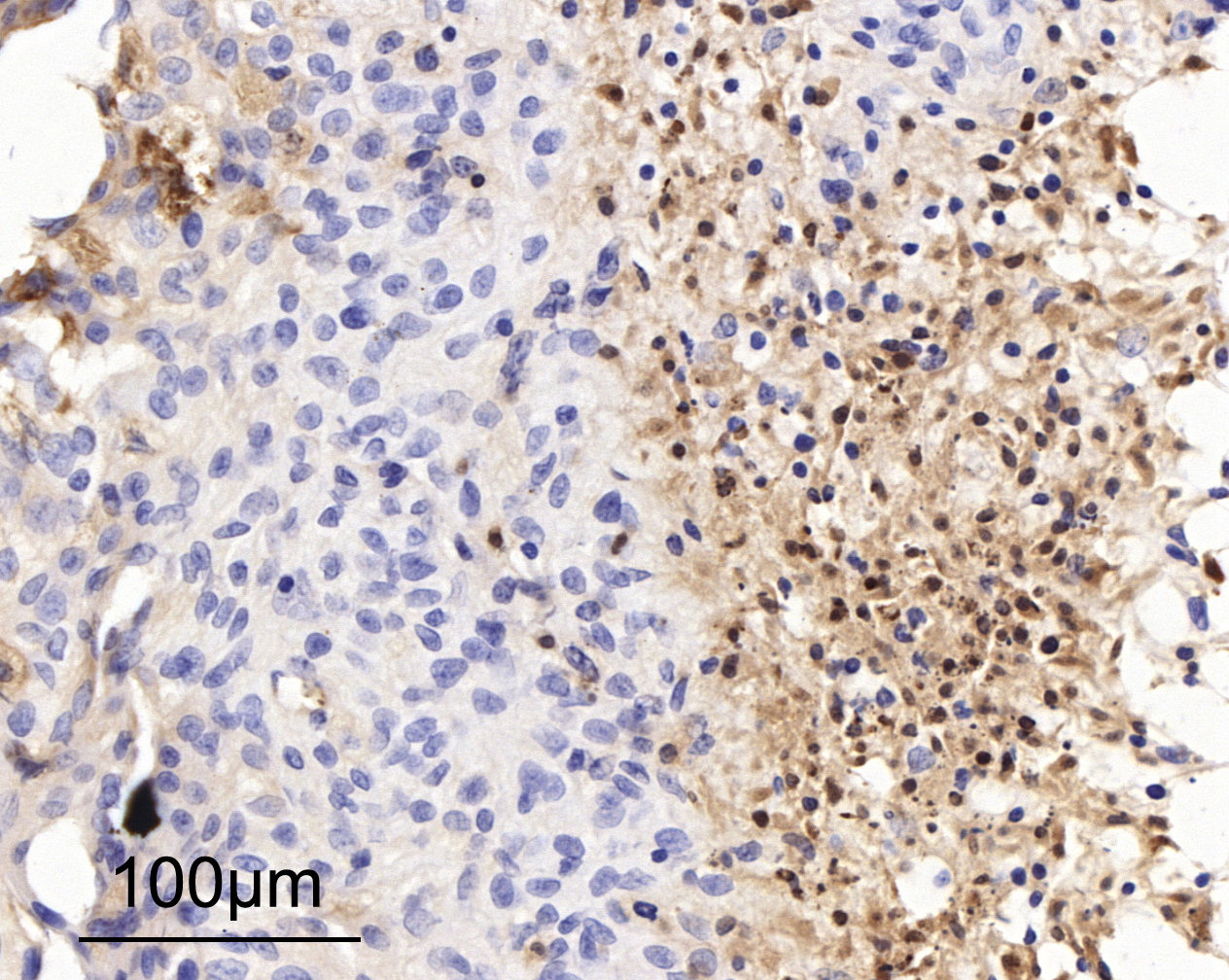

Supplement: Supplementary file 1 [file DataSheet1.ZIP › Original data/Figure 4/Figure 4G 3.jpg]

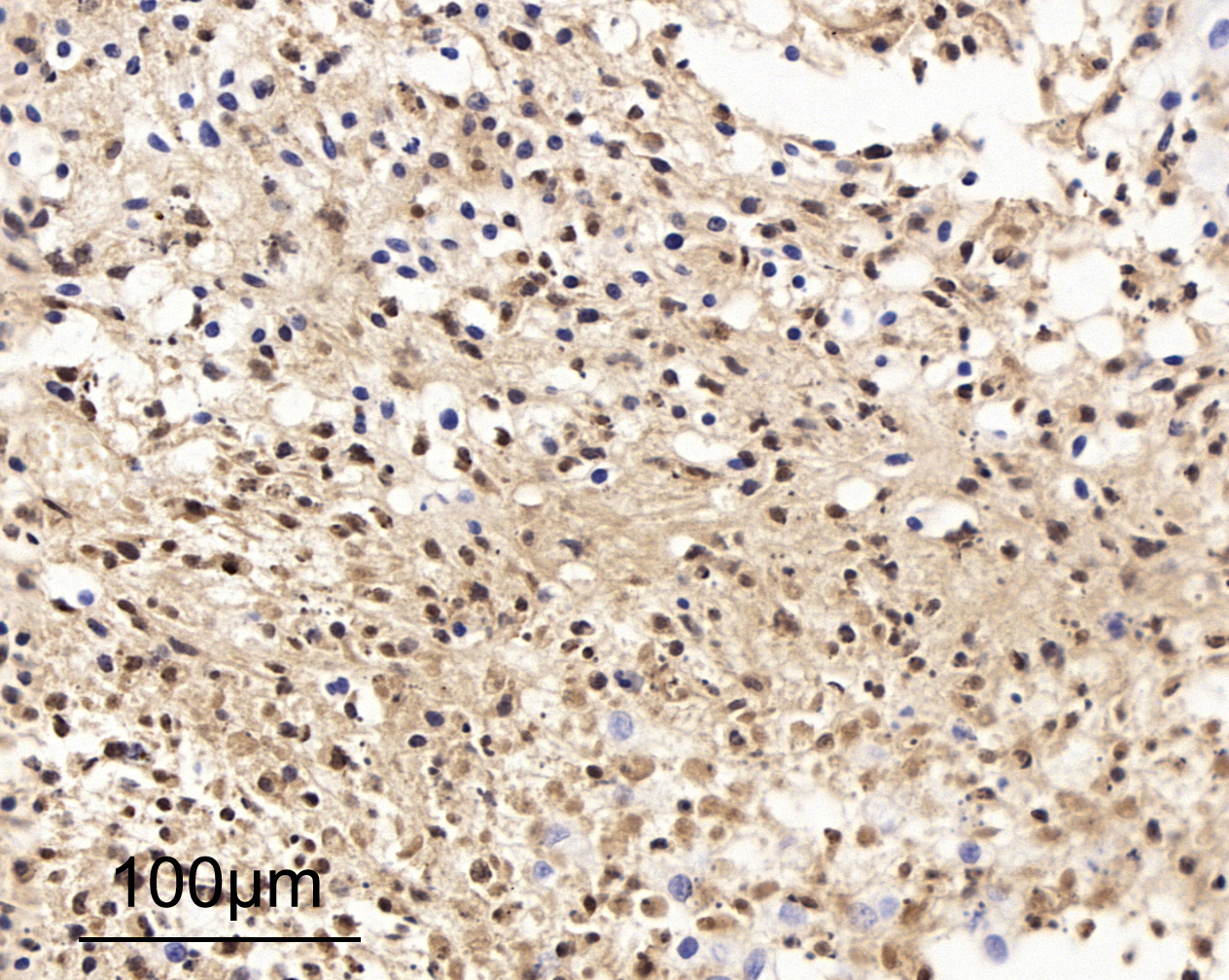

Supplement: Supplementary file 1 [file DataSheet1.ZIP › Original data/Figure 4/Figure 4G 4.jpg]

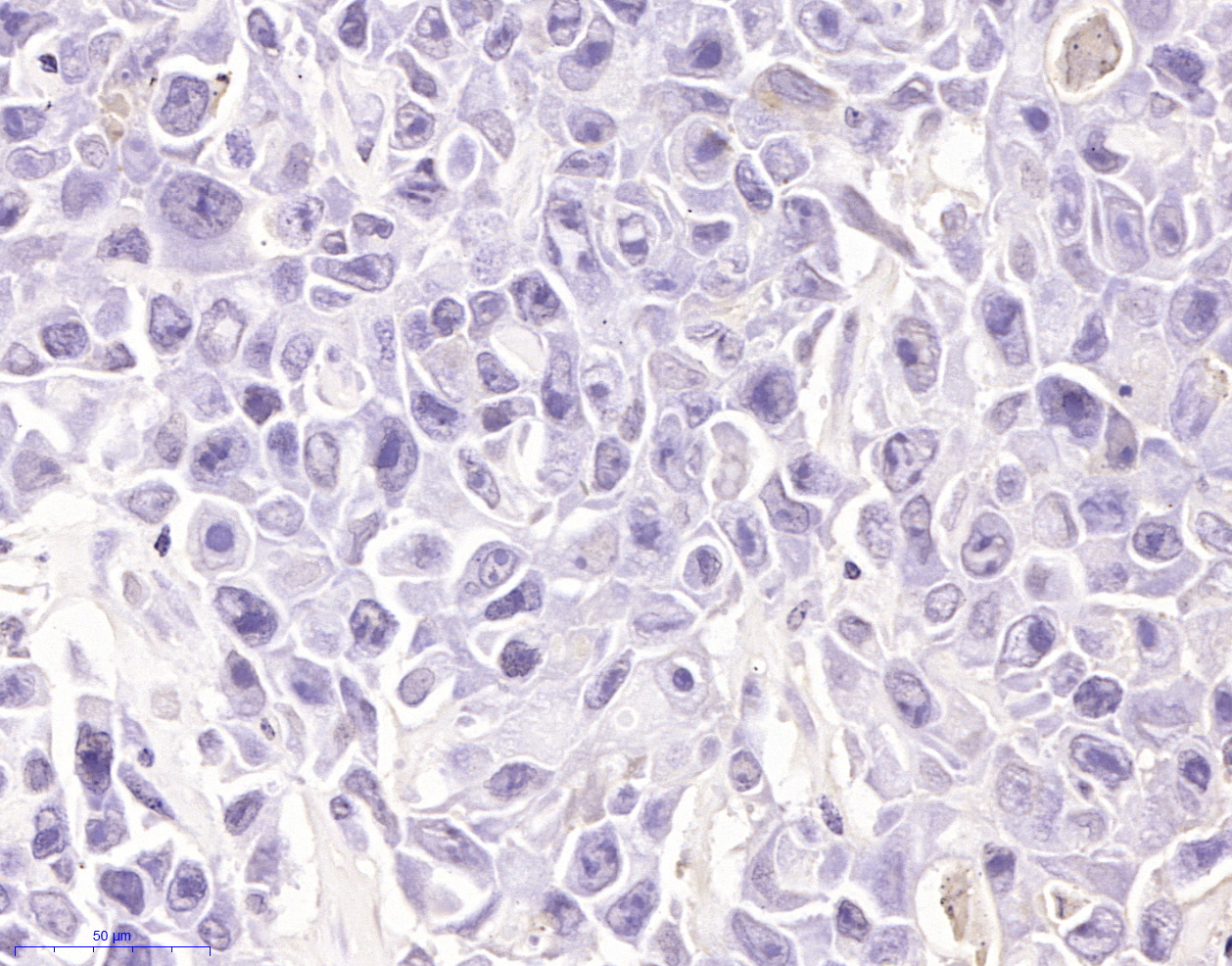

Supplement: Supplementary file 1 [file DataSheet1.ZIP › Original data/Figure 4/Figure 4H 1.jpg]

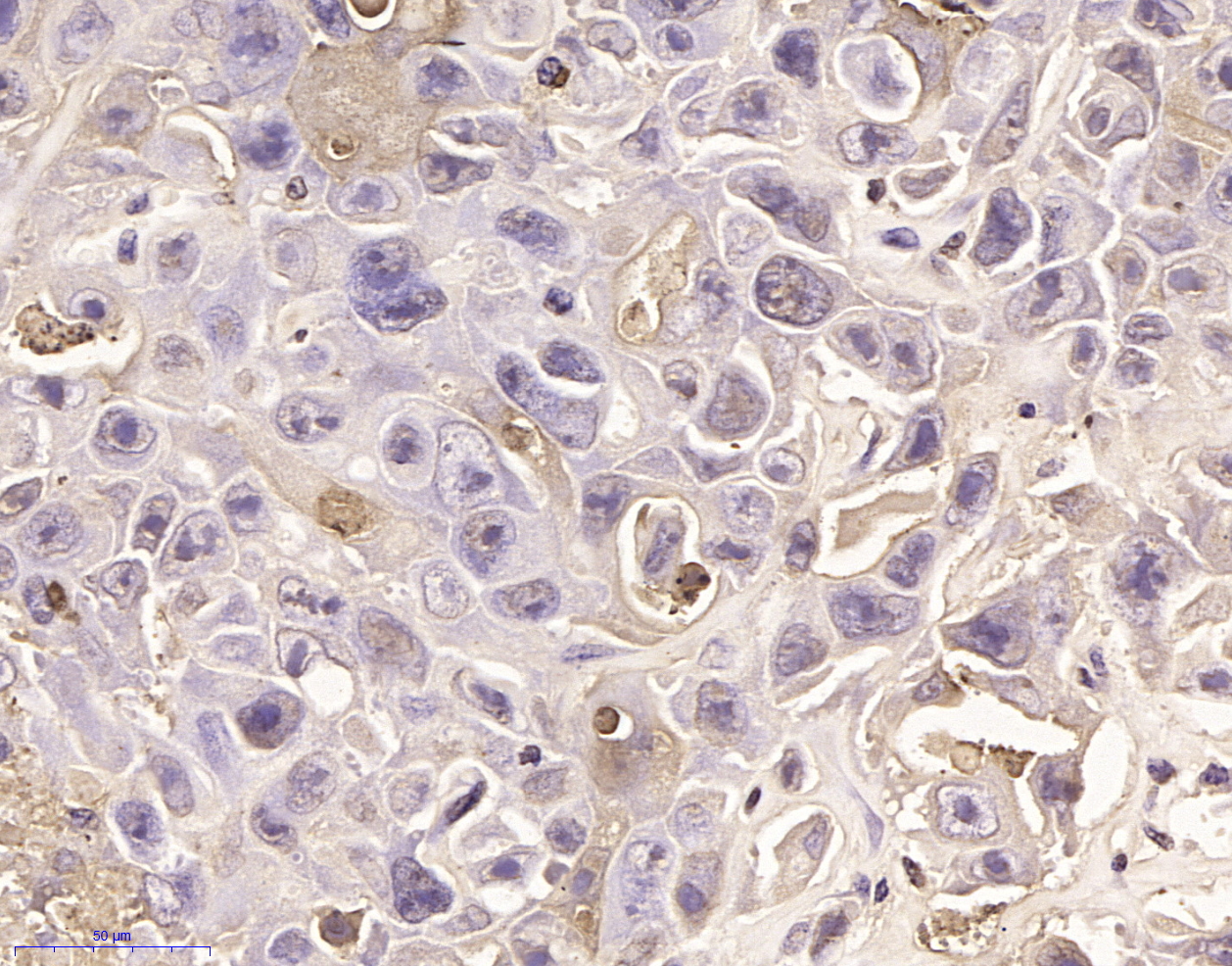

Supplement: Supplementary file 1 [file DataSheet1.ZIP › Original data/Figure 4/Figure 4H 2.jpg]

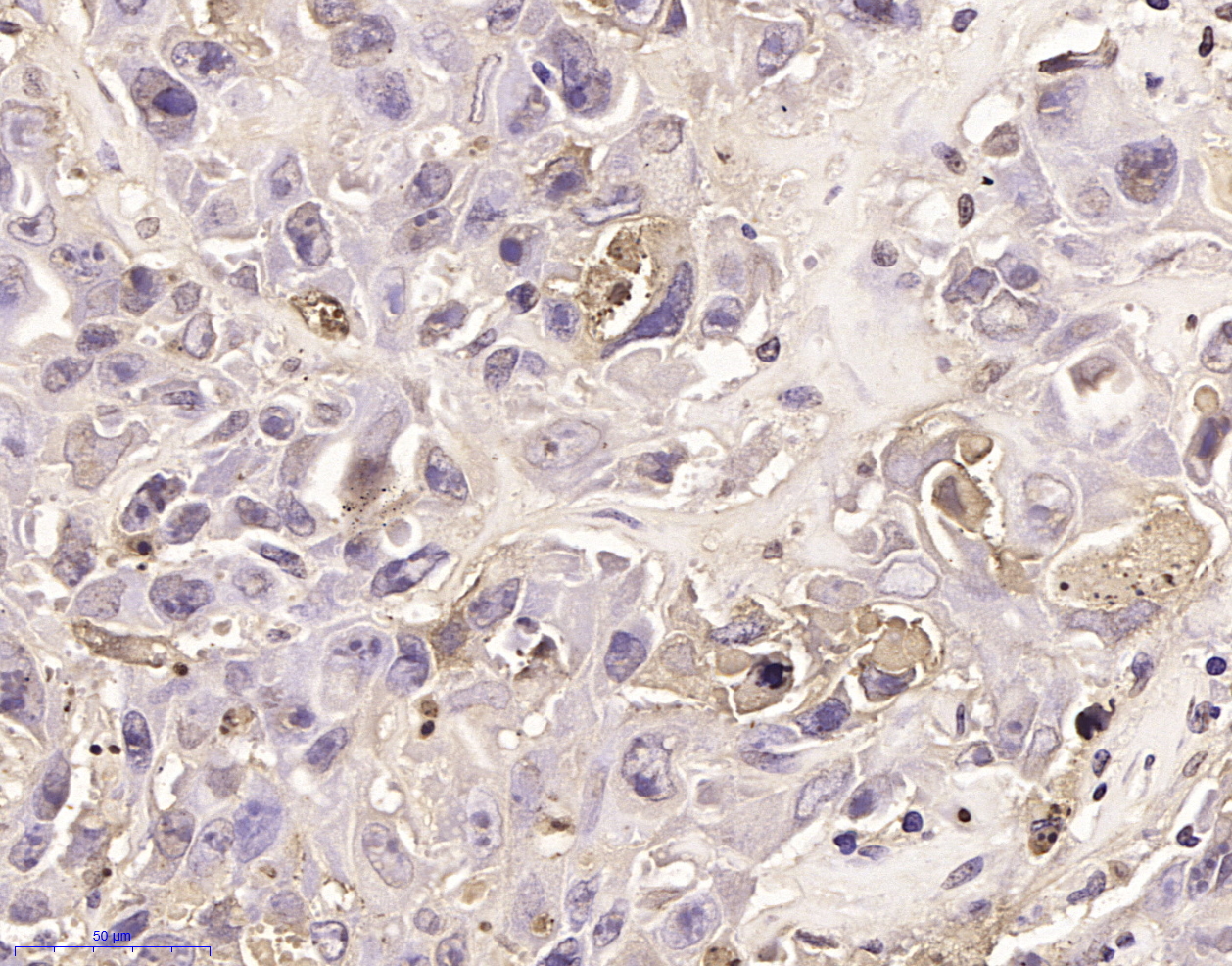

Supplement: Supplementary file 1 [file DataSheet1.ZIP › Original data/Figure 4/Figure 4H 3.jpg]

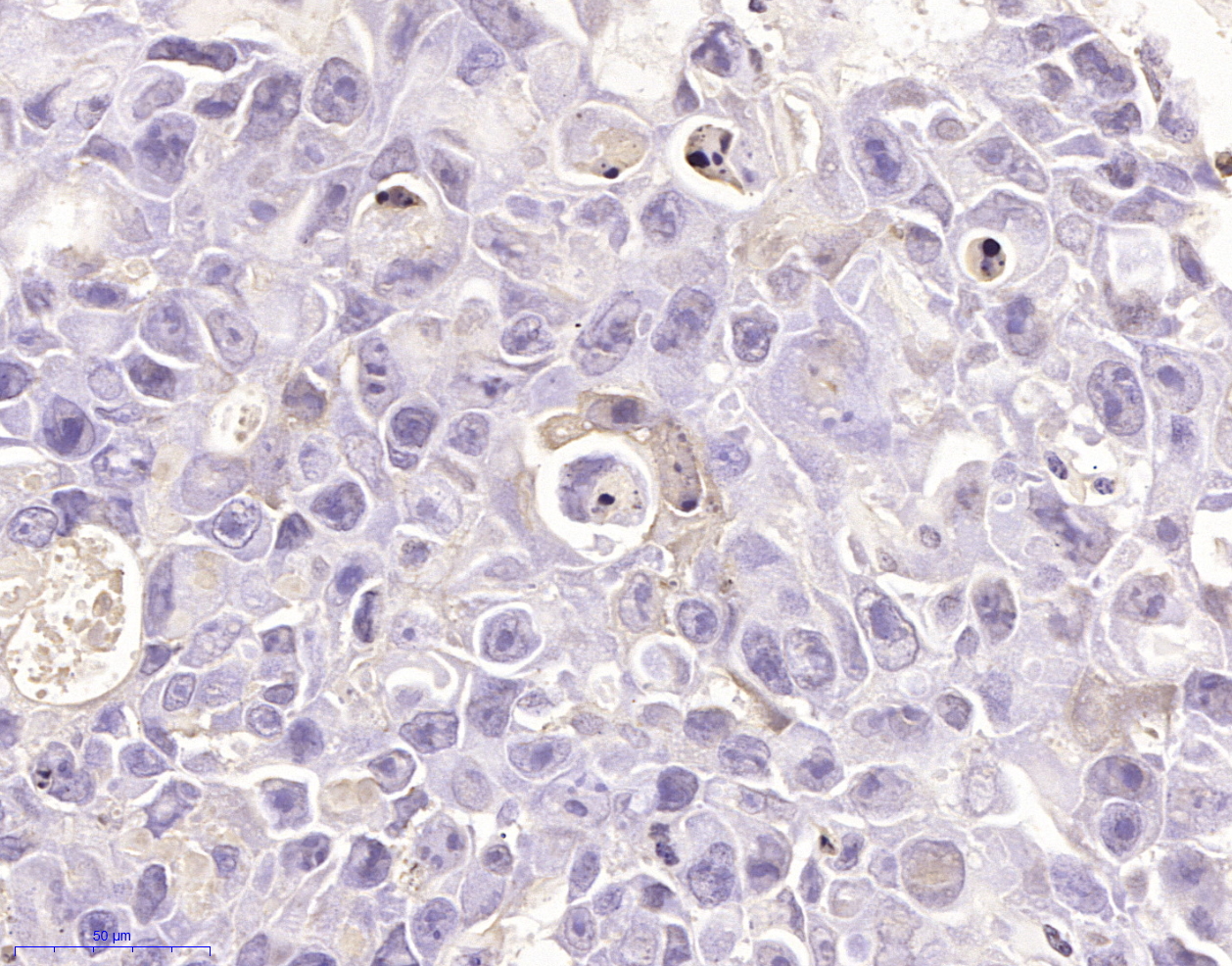

Supplement: Supplementary file 1 [file DataSheet1.ZIP › Original data/Figure 4/Figure 4H 4.jpg]

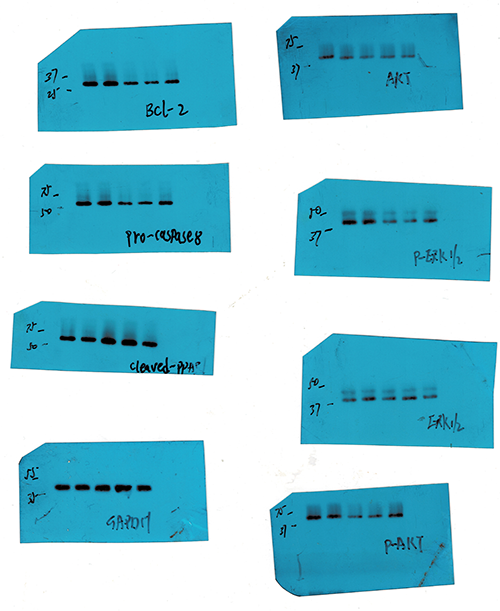

Supplement: Supplementary file 1 [file DataSheet1.ZIP › Original data/Figure 5/Figure 6A.tif]

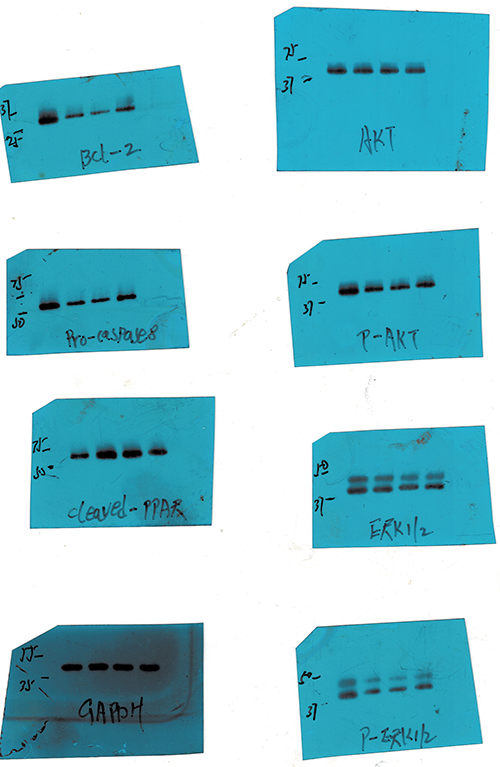

Supplement: Supplementary file 1 [file DataSheet1.ZIP › Original data/Figure 5/Figure 6B.tif]
